# Betrayal trauma and adult mental health: The role of mentalizing and dissociation

**DOI:** 10.1371/journal.pone.0353662

**Published:** 2026-07-13

**Authors:** Monika Olga Jańczak, Giulia Gagliardini, Anna Kamza, Antonello Colli, Peter Fonagy, Tobias Nolte

**Affiliations:** 1 Faculty of Psychology and Cognitive Science, Adam Mickiewicz University, Poznań, Poland; 2 Department of Humanities and Social Sciences, Universitas Mercatorum, Rome, Italy; 3 Institute of Psychology, Center for Research on Personality Development, SWPS University, Warsaw/Poznań, Poland; 4 Department of Humanities, University of Urbino “Carlo Bo”, Urbino, Italy; 5 Research Department of Clinical, Educational and Health Psychology, University College London, London, United Kingdom; 6 Anna Freud Centre, London, United Kingdom; The Hong Kong Polytechnic University, HONG KONG

## Abstract

**Background:**

Trauma experienced across the lifespan has been linked to a wide range of adverse mental health outcomes. However, the psychological mechanisms connecting betrayal trauma to later psychopathology remain insufficiently understood.

**Objective:**

This cross-sectional study investigated associations between betrayal trauma during childhood and adulthood and adult psychopathology—specifically depressive symptoms and level of personality functioning within a dimensional model of personality disorders. We tested a path model examining direct and indirect associations between betrayal trauma at different developmental periods and adult psychopathology via dissociation and hypomentalizing.

**Participants:**

A sample of 209 adults (61% female; aged 18–45 years; *M* = 29.7, *SD* = 7.88) were recruited from community and clinical settings in Poland.

**Methods:**

Participants completed validated self-report measures assessing betrayal trauma, depressive symptoms, personality functioning (ICD-11 model), dissociation, and mentalizing. Path analyses with parallel mediation were conducted using Satorra–Bentler estimation and 5,000 bootstrap resamples to examine both direct and indirect associations.

**Results:**

Both childhood and adulthood betrayal trauma were significantly associated with depressive symptoms and PD severity through dissociation and hypomentalizing [χ²(6) = 13.68, p = .033; CFI = 0.989; TLI = 0.931; RMSEA = 0.078; SRMR = 0.038]. No significant direct effects were observed once psychological processes were included. Mentalizing consistently demonstrated a stronger indirect association than dissociation across models. Adulthood betrayal trauma showed a greater total effect on depressive symptoms (β = .30; p < .001) than childhood trauma (β =  .17; p = .043), whereas their effects on personality pathology were comparable (respectively, β =  .21; p = .006 for adult trauma and β = .22; p = .008 for childhood trauma). For adulthood trauma and PD severity, the direct association was small and non-significant (β = −.06), whereas indirect effects via dissociation (β = .11) and mentalizing (β = .16) were positive, resulting in a positive total effect.

**Conclusions:**

These findings indicate that the link between betrayal trauma and adult psychopathology may be best conceptualised in terms of co-occurring psychological processes rather than direct exposure effects. Hypomentalizing, in particular, appears to represent a key transdiagnostic mechanism connecting relational trauma across developmental stages with both mood and personality pathology.

## Introduction

Trauma has been defined as the result of the interaction between a psychologically overwhelming event and an individual’s physical and mental reactions to that event [1]. Different forms of trauma have been delineated, each conferring distinct consequences for the affected person. Bateman and Fonagy [2] distinguish interpersonal forms of adversity—especially those occurring within attachment relationships—from more impersonal events, and highlight the impact of attachment and developmental trauma when the perpetrator is an attachment figure and the child’s developmental processes are implicated. Complementing this, betrayal trauma theory differentiates adverse experiences by the degree of betrayal involved: maltreatment perpetrated by a trusted other within a relationship of attachment or dependency constitutes a specific subtype of interpersonal trauma, distinct from harm inflicted by non-significant others [3,4]. Such experiences may entail unique adaptational demands. A central proposition is that defensive unawareness of abuse can serve short-term psychological protection when the perpetrator is a caregiver, yet such adaptations may undermine longer-term trust in others and in social information—i.e., produce epistemic disruption—and thereby diminish salutogenesis [5–7].

### Developmental mechanisms of trauma

Trauma may occur across different developmental periods, with both childhood and adulthood experiences contributing to later psychological functioning. Although childhood adversity is a well-established transdiagnostic risk factor for compromised developmental and mental health outcomes, it does not invariably lead to psychopathology. Empirical evidence highlights the heterogeneity of trajectories following adversity [8], encompassing chronic stress, delayed or immediate onset of symptoms, transient or persistent dysfunction, as well as resilience and post-traumatic growth [9–11]. In contrast, research on the impact of trauma occurring later in life—particularly during adolescence and adulthood—remains relatively scarce. While early interpersonal trauma has been linked to enduring vulnerabilities, trauma occurring in adulthood may represent more proximal stressors with direct implications for current symptomatology. Existing findings indicate that adolescent trauma can have enduring effects on psychosocial functioning, especially in the context of PD vulnerability [12–15]. In adulthood, trauma is most often examined in relation to (complex) PTSD or affective disorders such as anxiety and depression [16]. Compared with early-life trauma, adult-onset trauma is generally associated with less severe symptom complexity (e.g., [6,17,18]), potentially because it affects an already consolidated personality structure and is more likely to manifest in affective and symptomatic outcomes. By contrast, trauma occurring earlier in life may disrupt key developmental processes, leading to broader and more pervasive consequences for psychological functioning. Eearly and late trauma are often interrelated, partly due to processes such as revictimization [19], yet they may also show distinct associations with psychological outcomes, highlighting the importance of considering both developmental timing and cumulative exposure.

This developmental distinction is supported by neurodevelopmental research showing that the effects of trauma on brain structure and function vary depending on timing of exposure [20]. Early adversity has been linked to alterations in limbic and prefrontal systems involved in emotion regulation, whereas trauma occurring later in life may reflect different neuroplastic responses [21–23]. These findings suggest that trauma during sensitive developmental periods may disrupt core regulatory and representational capacities, whereas later trauma may have more context-dependent and symptom-related effects.

From a developmental psychopathology perspective, early childhood represents a particularly sensitive period during which trauma may have cascading effects across biological, cognitive, and relational domains, potentially disrupting the development of affect regulation and social-cognitive capacities [5,6,24–26]. By contrast, adults may be able to recruit higher-order regulatory processes, including more effective emotion regulation and mentalizing, which can mitigate the severity or expression of psychopathology. If trauma-related effects unfold as developmental cascades, their earlier manifestations may be observable in proximal cognitive–affective processes, such as dissociation and uncertainty about mental states (i.e., hypomentalizing), before becoming reflected in broader psychopathological outcomes. This cascade perspective therefore prioritises indirect mediatonal pathways over direct exposure–outcome links. Clarifying the mechanisms underlying these divergent developmental outcomes remains crucial to understanding the heterogeneity of trauma-related psychopathology—particularly in cases involving relational betrayal.

### Mentalizing and dissociation as protective and risk factors following trauma

A growing body of research has identified numerous mediators linking traumatic experiences to the emergence of psychopathology, among which ineffective mentalizing [1,26] and dissociation [27] are consistently supported. Mentalizing refers to the capacity to understand and interpret one’s own and others’ behaviours and experiences in terms of intentional mental states [2]. This is a multidimensional capacity that can be compromised in different ways. One manifestation is hypomentalizing, characterised by uncertainty about mental states and a reduced ability to infer and interpret the thoughts, feelings, and intentions underlying behaviour [28,29]. The literature reliably demonstrates associations between mentalizing and PD [30]. Ineffective mentalizing is considered a core mechanism underlying disturbances in emotion regulation, interpersonal functioning, and self-representation, which are central features of personality pathology [2]. More recently, research has increasingly focused on mentalizing within dimensional models of PD, including ICD-11 and the DSM-5 Alternative Model of Personality Disorders (AMPD) [31,32]. These findings suggest that impairments in mentalizing are systematically related to the severity of personality pathology, supporting a transdiagnostic understanding of mentalizing in personality disorders. Moreover, evidence from clinical populations suggests that mentalizing mediates the association between childhood maltreatment and severe psychiatric conditions, including PDs, major mood disorders, (complex) PTSD, and negative psychotic symptoms [33–36]. Mentalizing capacity develops within the context of attachment relationships, and substantial evidence indicates that childhood maltreatment undermines both the robustness and flexibility of this capacity [37]. The strength of this relationship varies according to the age of onset, type, and duration of trauma [6,18]. Cross-sectional and longitudinal studies consistently show that children exposed to maltreatment or neglect display ineffective mentalizing, which may represent adaptive adjustments to hostile or unpredictable developmental environments and later manifest as vulnerability to psychopathology [37–39]. Despite this substantial body of research, relatively little is known about the effects of trauma occurring later in life—particularly during late adolescence or adulthood—on mentalizing capacity and its relationship with mental health outcomes.

Dissociation has been widely conceptualised as a maladaptive response to early trauma (see meta-analytic reviews in [27,40]. This process appears to be particularly salient in the context of betrayal trauma, where the caregiver is also the perpetrator, creating an inescapable relational conflict [41,42]. In such situations, dissociation may serve as a psychological strategy that allows the child to manage overwhelming affect while maintaining dependence on the abusive caregiver for survival [41]. Dissociation in the context of betrayal trauma has been consistently linked to both personality pathology [43,44] and depressive symptoms [45, 46], with evidence drawn from both English-speaking and culturally diverse samples. Importantly, cross-cultural research suggests that the psychological impact of betrayal trauma is shaped by broader social and cultural contexts. For instance, Fung, Chien et al. [43] highlighted the mediating role of cultural resources and community support in the association between betrayal trauma and psychological outcomes, underscoring the importance of culturally sensitive interventions. Similarly, Gómez [47] and Huang et al. [48] showed that experiences of betrayal and their psychological consequences may manifest differently across cultural settings, calling for integrative and context-sensitive therapeutic approaches. Despite these advances, research on the psychological processes associated with betrayal trauma has focused mainly on English-speaking and, more recently, East Asian contexts, while evidence from Eastern European countries remains scarce. Addressing this gap is essential for advancing culturally informed models of trauma-related psychopathology.

Both mentalizing and dissociation are often construed as sequelae of trauma; yet while dissociation indexes a fragmentation of reflective capacity, mentalizing reflects an integrative function that may counteract such fragmentation—two sides of how the mind manages overwhelming relational threat [49]. Empirical work generally supports an inverse association: higher levels of dissociation correspond with less effective mentalizing, particularly in individuals with histories of adversity [50,51]. In their study of borderline PD severity and cPTSD, Bateman et al. [50] found that mentalizing partially mediated the relationship between dissociative symptoms and cPTSD but did not mediate the link between childhood abuse and cPTSD. Katzman and Papouchis [52] similarly reported that neither hypermentalizing nor hypomentalizing mediated the association between childhood trauma and dissociation; however, dissociation mediated the relationship between childhood trauma and hypermentalizing. Conversely, Wagner-Skacel et al. [51] observed that the association between adverse childhood experiences and dissociation was fully mediated by mentalizing. These mixed findings highlight the complexity of the relationship between the two constructs and underscore the need for further research that considers the timing of trauma exposure. As trauma occurring later in life may engage distinct dissociative and reflective mechanisms [53], elucidating these developmental contingencies is key to understanding the variability in post-trauma outcomes.

### Aims of the study

The primary aim of this study was to examine the roles of mentalizing and dissociation as key psychological processes linking relational adversity occurring at different developmental stages to adult psychopathology, with mentalizing conceptualized as a potential protective factor and dissociation as a risk factor for this relationship. Using a cross-sectional design, the study sought to: (a) examine the associations between betrayal trauma experienced in childhood versus adulthood and adult psychopathological outcomes (depression and level of personality functioning); and (b) investigate the indirect associations of mentalizing and dissociation, conceptualised as proximal outcomes of traumatic experiences, in these relationships. Given the paucity of research examining trauma in relation to the ICD-11 model of PD, a dimensional ICD-11 level of personality functioning was employed as a measure of PD. To capture variability across levels of symptom severity, the study adopts a dimensional approach, including participants from both clinical and community settings. Additionally, the study provides data from an underrepresented Eastern European context, extending evidence previously derived mainly from English-speaking and East Asian samples.

It was hypothesised that betrayal trauma occurring at different life stages would exhibit differential associations with psychopathology. Drawing on a developmental perspective as stated above, we assumed that betrayal trauma occurring earlier in life would be associated with more pervasive adult psychopathology, including both PD severity and depressive symptoms. In contrast, given that personality structure is largely consolidated by adulthood, trauma occurring later in life may function more as a proximal stressor linked to depressive symptoms than as a factor shaping enduring personality pathology.

It was further hypothesised that higher levels of betrayal trauma would correspond with greater  mentalizing difficulties (i.e., hypomentalizing) and higher dissociation, particularly for childhood trauma. Hypomentalizing and dissociation were expected to show positive associations with both depression and PD severity. Finally, consistent with the developmental psychopathology framework proposed by Bateman and Fonagy [2], it was anticipated that hypomentalizing would be positively correlated with dissociation. Given the mixed and bidirectional findings in prior research, and the lack of consistent evidence supporting a unidirectional pathway, the present study modelled dissociation and mentalizing as parallel mediators, capturing their potentially independent yet interrelated contributions to trauma-related psychopathology.

## Materials and methods

### Transparency and openness

All aspects of recruitment, data handling, and measurement are reported in accordance with the *Journal Article Reporting Standards* [54]. The dataset is publicly available at https://osf.io/976es/overview?view_only=64f52e6729b54ddfa48d6fe2b110067e

### Ethics and consent procedure

All participants provided written informed consent prior to taking part in the study. The consent process was conducted entirely in person. Participants received detailed information about the study objectives, procedures, and potential risks, which was presented both verbally and in written form. Only adult participants without cognitive impairments or disturbances in reality testing that could interfere with their capacity to provide informed consent were eligible for inclusion (see exclusion criteria below). The informed consent procedure was carried out by experienced clinical psychologists, who were responsible for ensuring that participants fully understood the nature of their participation. To support comprehension, a structured checklist was used to verify participants’ understanding of key study elements, including the purpose of the research, data protection and confidentiality, the voluntary nature of participation, the right to withdraw at any time without consequences, and the procedures involving specific research instruments. Following confirmation of understanding, participants signed a written consent form. All study procedures, including those related to the assessment and safeguarding of informed consent (e.g., inclusion and exclusion criteria, clarity of participant information materials, use of a comprehension checklist, and the qualifications of the research staff), were reviewed and approved by the Human Research Ethics Committee at Department of Psychology and Cognitive Science, Adam Mickiewicz University(Decision No. 07/06/2023).

### Participants and procedure

This cross-sectional study employed a paper-and-pencil design. Data were collected between 1 September 2023 and 20 April 2024. A total of 209 adults participated (61% female, 38% male, 1% other), aged 18–45 years (*M* = 29.7, *SD* = 7.88). Participants were recruited from two sources: (a) community-based volunteers via social media advertisements (*n* = 114) and (b) clinical participants from two psychiatric clinics in Poland (*n* = 94). Inclusion criteria were age (18–45) and fluency in Polish. The upper age limit was set to minimise recall bias, as younger and middle-aged adults are more likely to provide accurate retrospective reports than older individuals, whose recollections may be affected by age-related memory decline. Exclusion criteria included acute psychosis, schizoaffective or bipolar disorder, active substance dependence, brain injury or neurological disorder, and significant language difficulties.

A post-hoc power check following Fritz and MacKinnon [55] indicated adequate sensitivity to detect medium indirect effects in our parallel mediation: the required *n* ≈ 126 for *a* = *b* = .30, whereas our sample (*N* = 209) exceeded this threshold by ~66%.

## Measures

All constructs were assessed using standardised self-report instruments with established psychometric properties.

**Brief Betrayal Trauma Survey (BBTS;** [3]) The BBTS is a 14-item measure assessing exposure to major traumatic events across different life stages: before age 12 (childhood), ages 12–17 (adolescence), and after age 18 (adulthood). Traumas are classified as *low-betrayal* (minimal violation of trust) or *high-betrayal* (severe violation of trust by a close person). Items are rated on a three-point scale: 0 (*never*), 1 (*once or twice*), and 3 (*many times*). Only childhood and adulthood indices were used in this study. A Polish translation of the BBTS was developed using a multi-step procedure. Three independent forward translations were prepared by experienced clinicians with advanced proficiency in English, followed by expert panel reconciliation. The reconciled version was then back-translated into English by an independent translator experienced in academic translation and compared with the original instrument to ensure semantic and conceptual equivalence. The final version was reviewed in consultation with the original author of the instrument (J. Freyd). A more detailed description of the adaptation and validation process is provided elsewhere [56]. The BBTS has demonstrated good construct validity and test–retest reliability [3]. As a checklist measure, internal consistency is not reported.

**Patient Health Questionnaire (PHQ-9**; [57]) The PHQ-9 is a widely used screening measure for depressive symptoms, comprising nine items rated on a four-point scale from 0 (*not at all*) to 3 (*nearly every day*). Total scores range from 0 to 27, with higher scores indicating greater symptom severity. Internal consistency in the current sample was good (Cronbach’s α = 0.89).

**Self and Interpersonal Functioning Scale (SIFS;** [58]) The SIFS, adapted to Polish by Soroko et al. [59], assesses dimensional aspects of personality pathology consistent with the ICD-11 framework [60]. The 24-item measure includes four subscales: identity (α = 0.84), self-direction (α = 0.77), empathy (α = 0.73), and intimacy (α = 0.75). The SIFS has demonstrated sound validity and clinical utility in capturing personality functioning across both community and clinical populations.

**Dissociative Experiences Scale–Revised (DES-R;** [61]) The DES-R measures dissociative experiences using 28 items rated on an eight-point scale (0 = *never* to 7 = *once a day or more often*). It assesses both pathological symptoms (e.g., amnesia, intrusive traumatic memories, pseudohallucinations) and non-pathological absorption states. The Polish version [62] has demonstrated good psychometric properties. Internal consistency in the current sample was excellent (Cronbach’s α = 0.96).

**Reflective Functioning Questionnaire (RFQ-8;** [63]) The RFQ-8 is an eight-item self-report measure of mentalizing. Each item is rated on a seven-point Likert scale (1 = *strongly disagree* to 7 = *strongly agree*). The questionnaire includes two subscales: certainty (RFQ_C) and uncertainty (RFQ_U) about mental states. In the present study, we focused on the RFQ_U subscale, which captures uncertainty about mental states (i.e., hypomentalizing). This decision was based on prior psychometric evidence indicating that RFQ_U demonstrates more robust properties and clearer interpretability compared to RFQ_C, which has been questioned as a valid indicator of ineffective mentalizing (e.g., [64,65]). In addition, focusing exclusively on RFQ_U avoids methodological issues related to double-scoring and the mathematical dependence between the two subscales, while remaining consistent with more recent unidimensional approaches to RFQ scoring  [64,65]. This approach has also been adopted in recent studies using the RFQ (e.g., Bateman et al., 2023). The validated Polish version [65] was used and demonstrated acceptable internal consistency in the present sample (Cronbach’s α = 0.79).

### Statistical analyses

The proportion of missing data was low (1.10% overall), with no clear systematic pattern of missingness observed across variables. Missing data were imputed using the *missForest* algorithm, a non-parametric method based on random forests that iteratively predicts missing values from observed data without assuming variable normality. This approach minimises bias and provides robust estimates even in complex data structures. Descriptive statistics were calculated for all variables. Given that formal normality tests are overly sensitive in large samples, univariate normality was assessed via skewness and kurtosis values, with values between –2 and +2 considered acceptable. All variables met this criterion. Associations between key study variables and sociodemographic characteristics were examined using Pearson’s correlation coefficients for continuous variables (age, childhood betrayal trauma, adulthood betrayal trauma, dissociation, mentalizing, depressive symptoms, and PD severity), Spearman’s rho for the ordinal variable (education), and point-biserial correlations for binary variables (sex, marital status). Correlation coefficients were interpreted as follows: 0–.30 as weak, .30–.70 as moderate, and .70–1.0 as strong relationships.

A path analysis with a parallel multiple mediator model was conducted to examine patterns of direct and indirect associations linking childhood and adulthood betrayal trauma with depressive and PD symptoms, with dissociation and hypomentalizing specified as mediators. Given the cross-sectional design, mediation was tested as statistical indirect effects in a path-analytic model rather than as evidence of causal mechanisms. Demographic covariates (age, education, and marital status) were included as control variables through covariances with endogenous variables. The model also specified covariances between mediators (dissociation and mentalizing), between outcome variables (depression and PD symptoms), and between the two trauma indices (childhood and adulthood). In the first mediational pathway, direct paths were modelled from both trauma variables to dissociation, and from dissociation to both outcome variables. In the second pathway, direct paths were specified from both trauma variables to hypomentalizing, and from hypomentalizing to each outcome. This structure allowed for the decomposition of total effects into direct and specific indirect effects via each mediator. Recruitment source (clinical vs. community) was examined in additional exploratory analyses as a potential moderator of the associations between betrayal trauma and the mediators.

Analyses employed Satorra–Bentler robust estimation [66] to account for potential non-normality, with all fit indices except SRMR incorporating this correction. The stability of indirect effects was assessed using a bootstrap procedure with 5,000 resamples, providing bias-corrected 95% confidence intervals that do not rely on normality assumptions [67]. Effects were considered statistically significant when confidence intervals excluded zero. Model fit was evaluated using multiple indices in line with standard recommendations [68]. A non-significant chi-square statistic (*p* > .05) indicated good fit, though this index is sensitive to sample size. Incremental fit indices included CFI and TLI (>.95 good fit, > .90–.95 acce*p*table fit) and absolute fit indices included RMSEA (<.05 good fit, .05–.08 acceptable fit, > .10 poor fit); and the SRMR (<.08 satisfactory). Model parsimony was assessed using AIC and BIC, with lower values indicating superior fit while penalising complexity [69]. Standardised path coefficients (β) were interpreted using Cohen’s conventions, adapted for structural equation modelling: < .10 as small, ≈ .30 as medium, and >.50 as large effects. We followed Preacher and Hayes [70], to estimate relative contribution of each mediation pathway, expressed as the proportion of the total and indirect effects, respectively. Patterns consistent with statistical suppression (i.e., opposing signs of direct and indirect effects or indirect effects exceeding the total effect; [67]) were noted descriptively and interpreted with caution, particularly in cases where direct effects were small or non-significant.

Mediation proportions above 50% were interpreted as dominant mechanisms, 20–30% as substantial contributions, > 60% as primary mediators, and 40–60% as parallel processes. These thresholds were interpreted in light of theoretical considerations rather than as fixed cut-offs [71]. Explained variance (*R²*) values for endogenous variables were interpreted according to Ferguson’s [72] guidelines: .04 as the minimum practically significant effect, .25 as moderate, and .64 as strong. All analyses were performed in *R* (version 4.3.1) using the *lavaan* package (version 0.6–19).

## Results

### Descriptive statistics

Descriptive statistics and bivariate correlations among all study variables are presented in Table 1. Sex showed weak positive correlations with age and education (women were slightly older and more educated). Marital status correlated weakly negatively with childhood trauma, dissociation, hypomentalizing, depressive symptoms, and PD severity, and moderately positively with education. Age correlated moderately positively with education and weakly negatively with dissociation, depressive symptoms, and PD severity. Education correlated weakly negatively with childhood and adulthood betrayal trauma and moderately negatively with dissociation, hypomentalizing, depressive symptoms, and PD severity. Childhood betrayal trauma was moderately positively correlated with adulthood betrayal trauma, dissociation, hypomentalizing, depressive symptoms, and PD severity. Similarly, adulthood betrayal trauma showed moderate positive correlations with dissociation, hypomentalizing, depressive symptoms, and PD severity. Dissociation demonstrated moderate positive associations with hypomentalizing, depressive symptoms, and PD severity. Hypomentalizing was moderately correlated with depressive symptoms and strongly correlated with PD severity. Finally, a strong positive correlation was observed between depressive symptoms and PD severity.

**Table 1:** Descriptive statistics for the study variables (N = 209).

Variable	*M*	*SD*	Skewness (SE)	Kurtosis (SE)	1	2	3	4	5	6	7	8	9
1. Sex ^a, c^													
2. Marital status ^b, c^					.13								
3. Age ^d^	29.73	7.88	0.47 (0.17)	−0.62 (0.33)	.17*	.06							
4. Education ^e, f^	2.98	1.20	0.05 (0.17)	−1.19 (0.33)	.26***	.42***	.31***						
5. Childhood trauma ^d^	3.20	2.79	1.49 (0.17)	1.5 (0.33)	−.01	−.19**	−.06	−.27***					
6. Adulthood trauma ^d^	2.95	2.09	1.24 (0.17)	0.94 (0.33)	−.02	−.12	.11	−.27***	.53***				
7. Dissociation ^d^	37.76	24.33	0.4 (0.17)	−1.0 (0.33)	−.08	−.22**	−.27***	−.44***	.39***	.41***			
8. Hypomentalizing ^d^	5.72	4.48	1.03 (0.17)	0.42 (0.33)	.02	−.28***	−.03	−.34***	.36***	.43***	.51***		
9. Depression ^d^	11.79	7.07	0.52 (0.17)	−0.79 (0.33)	−.03	−.19**	−.23***	−.47***	.35***	.38***	.57***	.62***	
10. PDs ^d^	101.62	58.58	−0.02 (0.17)	−1.2 (0.33)	.15	−.28***	−.15*	−.43***	.35***	.33***	.61***	.70***	.72***

a -1 = female and 1 = male.

b -1 = single and 1 = in romantic relationship.

c Binary variables – Point – biserial correlation.

d Continuous variables - Pearson’s *r* coefficient.

e Ordinal sociodemographic variables - Spearman’s *rho* coefficient.

f Education and binary variables (Sex, Marital status) – *V*-Kramer’s coefficient.

* *p* < .05, ** *p* < .01, *** *p* < .001

### Indirect pathways linking betrayal trauma and psychopathology

**Fig 1:**
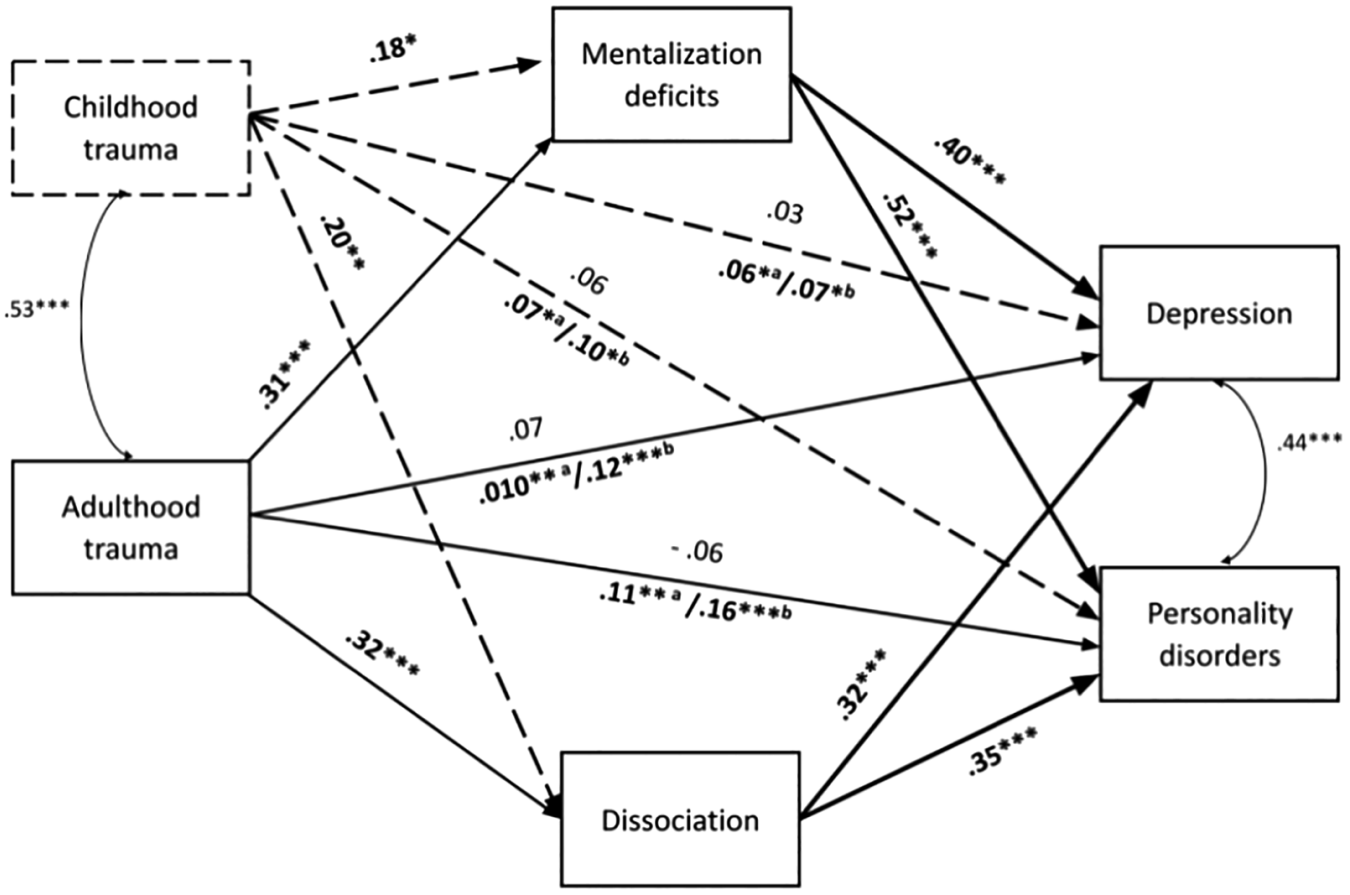
Path-analytic model depicting indirect associations between childhood and adulthood betrayal trauma and depressive symptoms and personality disorder (PD) severity through dissociation and hypomentalizing. Note. Path analysis results are shown. Values above arrows represent direct effects; those below arrows indicate indirect effects (via dissociation before slash, via hypomentalizing after). Dashed lines represent Childhood trauma effects, solid lines — Adulthood trauma effects. Paths from mediators to outcomes are solid, reflecting shared mechanisms. For clarity of presentation, residuals, error terms, control variable paths, and the covariance between mediators (β = .39) are omitted. The model controls for age, education, and marital status by incorporating relevant covariances with endogenous variables. Estimates are based on 5,000 bootstrap resamples.

The parallel mediation model tested whether betrayal trauma experienced in childhood and adulthood predicted depressive symptoms and PD severity directly and indirectly through dissociation and hypomentalizing. Age, education, and marital status were included as covariates. The model was evaluated using two estimation methods: Satorra–Bentler corrected maximum likelihood (MLM) and bootstrapping with 5,000 resamples. Both approaches produced nearly identical and satisfactory fit indices: χ²(6) = 13.68, *p* = .033; CFI = 0.989; TLI = 0.931; RMSEA = 0.078; SRMR = 0.038. Comparative information criteria were also consistent across methods (AIC = 11,109.588; BIC = 11,239.939). The high convergence between estimation approaches supports the robustness of the model and indicates that the proposed structure accurately captures the relationships among variables. As illustrated in Fig 1, the model explained 46.22% of the variance in depressive symptoms and 57.04% of the variance in PD severity—moderate to substantial effects according to Ferguson’s (2009) benchmarks. Both Satorra–Bentler robust estimation and bootstrap procedures produced congruent estimates, confirming the stability and reliability of the findings.

Table 2 presents the standardised direct and indirect effects of childhood and adulthood betrayal trauma on psychopathological outcomes via dissociation and hypomentalizing, with a comparison between Satorra–Bentler and bootstrap estimates, with bias-corrected percentile 95% confidence intervals derived from bootstrapping. Both childhood and adulthood betrayal trauma were significantly associated with depressive symptoms and PD severity primarily through indirect pathways. For depressive symptoms, both dissociation and hypomentalizing emerged as significant mediators. Childhood betrayal trauma showed weak positive indirect effects on depression via both dissociation and mentalizing, yielding an overall weak positive total effect. Adulthood betrayal trauma demonstrated weak-to-moderate indirect effects through both mediators, resulting in a relatively stronger total positive effect. Neither childhood nor adulthood betrayal trauma exerted significant direct effects on depressive symptoms, indicating indirect effects in the absence of significant direct effects. These findings suggest that betrayal-related experiences contribute to depressive symptomatology largely through psychological processes of dissociation and hypomentalizing, which may heighten vulnerability to affective dysregulation. The stronger total effects observed for adulthood trauma imply that more recent betrayal experiences may exert a greater overall influence on current depressive symptoms than earlier trauma. For PD severity, both mediators again played significant roles. Childhood betrayal trauma had weak positive indirect effects through dissociation and hypomentalizing, resulting in a weak positive total effect. Adulthood betrayal trauma exhibited a weak indirect effect via dissociation and a moderately stronger indirect effect via hypomentalizing, producing weak-to-moderate total effects. As with depression, neither childhood nor adulthood betrayal trauma showed significant direct effects on personality pathology.

**Table 2:** Parallel Mediation Model: Comparison of Direct, Indirect, and Total Effects: Satorra-Bentler (N = 209) vs. Bootstrap Methods (5000 Resamples).

		Satorra-Bentler					Bootstrap					
Type	Path	*B*	β	*SE*	*z*	*p*	*B*	β	*SE*	*z*	*p*	95% *CI*
Paths to Mediators	Childhood trauma → Dissociation	1.71	0.20	0.62	2.76	.006	1.71	0.20	0.64	2.68	.007	[0.437, 2.921]
Paths to Mediators	Childhood trauma → Mentalization	0.29	0.18	0.13	2.30	.022	0.29	0.18	0.13	2.26	.024	[0.043, 0.543]
Paths to Mediators	Adulthood trauma → Dissociation	3.66	0.32	0.87	4.23	<.001	3.66	0.32	0.89	4.10	<.001	[1.921, 5.349]
Paths to Mediators	Adulthood trauma → Mentalization	0.65	0.31	0.16	4.04	<.001	0.65	0.31	0.17	3.92	<.001	[0.330, 0.976]
Paths from Mediators	Dissociation → Depression	0.09	0.32	0.02	4.79	<.001	0.09	0.32	0.02	4.64	<.001	[0.052, 0.130]
Paths from Mediators	Mentalization → Depression	0.64	0.40	0.11	5.77	<.001	0.64	0.40	0.12	5.54	<.001	[0.398, 0.850]
Paths from Mediators	Dissociation → PD	0.84	0.35	0.14	6.20	<.001	0.84	0.35	0.14	6.10	<.001	[0.558, 1.096]
Paths from Mediators	Mentalization → PD	6.83	0.52	0.62	11.07	<.001	6.83	0.52	0.64	10.61	<.001	[5.621, 8.119]
Direct Effects	Childhood trauma → Depression	0.08	0.03	0.17	0.49	.622	0.08	0.03	0.18	0.48	.634	[-0.251, 0.434]
Direct Effects	Childhood trauma → PD	1.25	0.06	1.27	0.99	.322	1.25	0.06	1.35	0.93	.352	[-1.204, 4.061]
Direct Effects	Adulthood trauma → Depression	0.25	0.07	0.22	1.13	.260	0.25	0.07	0.23	1.08	.279	[-0.207, .703]
Direct Effects	Adulthood trauma → PD	−1.71	−0.06	1.72	−1.00	.318	−1.71	−0.06	1.84	−0.93	.352	[-5.272, .999]
Indirect Effects via Dissociation	Childhood trauma → Depression	0.16	0.06	0.07	2.32	.020	0.16	0.06	0.07	2.25	.025	[0.044, 0.323]
Indirect Effects via Dissociation	Childhood trauma → PD	1.43	0.07	0.55	2.58	.010	1.43	0.07	0.58	2.48	.013	[0.442, 2.701]
Indirect Effects via Dissociation	Adulthood trauma → Depression	0.34	0.10	0.11	3.10	.002	0.34	0.10	0.12	2.81	.005	[0.149, 0.629]
Indirect Effects via Dissociation	Adulthood trauma → PD	3.07	0.11	0.95	3.24	.001	3.07	0.11	0.99	3.08	.002	[1.470, 5.481]
Indirect Effects via Mentalization	Childhood trauma → Depression	0.19	0.07	0.09	2.12	.034	0.19	0.07	0.09	2.06	.039	[0.035, 0.392]
Indirect Effects via Mentalization	Childhood trauma → PD	1.98	0.09	0.86	2.30	.022	1.98	0.09	0.88	2.24	.025	[0.318, 3.775]
Indirect Effects via Mentalization	Adulthood trauma → Depression	0.42	0.12	0.12	3.61	<.001	0.42	0.12	0.12	3.50	<.001	[0.220, 0.702]
Indirect Effects via Mentalization	Adulthood trauma → PD	4.43	0.16	1.13	3.93	<.001	4.43	0.16	1.16	3.81	<.001	[2.336, 6.950]
Total Effects	Childhood trauma → Depression	0.43	0.17	0.21	2.02	.043	0.43	0.17	0.22	1.98	.047	[0.013, 0.865]
Total Effects	Childhood trauma → PD	4.66	0.22	1.78	2.61	.009	4.66	0.22	1.86	2.51	.012	[1.014, 8.369]
Total Effects	Adulthood trauma → Depression	1.00	0.30	0.26	3.81	<.001	1.00	0.30	0.27	3.69	<.001	[0.459, 1.519]
Total Effects	Adulthood trauma → PD	5.79	0.21	2.13	2.71	.007	5.79	0.21	2.29	2.53	.011	[1.365, 10.391]

*Note*. PD = personality disorders.

Bootstrap 95% confidence intervals are bias-corrected.

All significant indirect effects operate via mentalizing; direct effects are non-significant, indicating indirect-only mediation pattern.

### Relative contributions of indirect pathways

To assess the relative influence of each mediator in the relationship between betrayal trauma and psychopathology, the proportion of the total effect mediated by each pathway and each pathway’s contribution to the total indirect effect were calculated, following Preacher and Hayes [70]. This approach provides a nuanced understanding of the comparative strength of each mediating mechanism beyond significance testing alone.

The analysis of relative contributions underscored the dominant role of indirect effects across all trauma–outcome relationships, which accounted for between 73.11% and 129.62% of total effects (see Table 3). This finding highlights the central importance of the mediating mechanisms—dissociation and hypomentalizing—in explaining the associations between betrayal trauma and psychopathology. The single value exceeding 100% (observed in the pathway from adulthood betrayal trauma to PD severity) reflects a statistical artefact arising from a near-zero and oppositely signed direct effect (β = –.06, ns) combined with a substantial positive indirect effect (β = .27, p < .001). Such cases can produce proportions exceeding 100% and should not be interpreted as evidence of meaningful suppression, but rather as indicating that the total effect is almost entirely carried by indirect pathways. Hypomentalizing consistently demonstrated stronger mediating effects than dissociation, accounting for 54.01–59.11% of the total indirect effects across all models, whereas dissociation accounted for 40.89–45.99%. This consistent pattern suggests that difficulties in understanding and interpreting mental states play a more central role than dissociative processes in linking betrayal trauma to both depressive and PD symptoms. Notably, the relationship between adulthood betrayal trauma and PD severity was characterised by a non-significant negative direct association (β = –.06) alongside positive indirect effects via mentalizing (β = 0.16) and dissociation (β = 0.11), resulting in a positive total effect (β = 0.21). This pattern indicates that the association operates almost entirely through these mediating mechanisms rather than directly. For adulthood betrayal trauma, indirect pathways accounted for 75.12% of the total effect on depressive symptoms and essentially all of the association with PD severity. Values exceeding 100% arise because the direct effect is very small and does not contribute meaningfully to the total effect.

**Table 3:** Path-analytic model: Relative contributions of direct and indirect effects in trauma-outcome relationships.

Trauma type	Outcome	% of total effect				% of indirect effect	
		Direct	*via* dissociation	*via* mentalization	Total Indirect	*via* dissociation	*via* mentalization
Childhood	Depression	19.53%	37.01%	43.46%	80.47%	45.99%	54.01%
Childhood	PD	26.89%	30.71%	42.40%	73.11%	42.01%	57.99%
Adulthood	Depression	24.88%	33.69%	41.43%	75.12%	44.85%	55.15%
Adulthood	PD	−29.62%	53.00%	76.62%	129.62%	40.89%	59.11%

*Note*. PD = PD symptoms

For childhood betrayal trauma, indirect pathways explained 80.47% of the total effect on depressive symptoms and 73.11% on PD severity. Overall, the consistent predominance of hypomentalizing across all models reinforces its importance as a key mechanism linking betrayal trauma to psychopathology.

### Covariances

The covariance analysis indicated several significant interrelations (see S1 Table). Depressive symptoms covaried moderately with PD severity; dissociation with hypomentalizing; and childhood with adulthood betrayal trauma. Among demographics, negative covariances predominated: age showed a moderate negative covariance with dissociation and a small negative covariance with depressive symptoms. Education showed a moderate negative covariance with dissociation and small negative covariances with childhood and adulthood betrayal trauma, hypomentalizing, depressive symptoms, and PD severity. Marital status showed weak negative covariances with dissociation and hypomentalizing, and a small negative covariance with childhood betrayal trauma. Additional exploratory analyses indicated that recruitment source (clinical vs. community) did not significantly moderate the associations between trauma and the mediators, and inclusion of interaction terms did not improve model fit (see Table S2 in the Supplementary Materials).

## Discussion

The primary aim of this study was to deepen understanding of the role of mentalizing and dissociation as markers of both protective and risk factors in the context of relational adversity across different life stages. Specifically, we examined how betrayal trauma—occurring in childhood or adulthood—relates to two core domains of adult psychopathology: depressive symptoms and PD severity. Consistent with our hypotheses, both hypomentalizing and dissociation emerged as significant indirect pathways linking betrayal trauma to psychopathological outcomes. The model accounted for 46.22% of the variance in depressive symptoms and 57.04% in PD severity, indicating substantial explanatory power. Importantly, by employing a dimensional measure of personality pathology aligned with the ICD-11 framework, this study extends the growing literature on the developmental roots of PD [73, 74], offering empirical support for the inclusion of trauma-related psychological processes within dimensional conceptualisations of PD. It also supplements previous cross-cultural studies on betrayal trauma and adult psychopathology by adding evidence from an Eastern European context, further supporting the cross-cultural robustness of these associations.

The absence of significant direct effects of betrayal trauma—whether in childhood or adulthood—on depressive and PD supports a model in which trauma relates to psychopathology primarily through indirect psychological pathways rather than through direct exposure effects. Hypomentalizing and heightened dissociation consistently accounted for these indirect effects across both developmental periods and outcome domains, indicating that the way trauma is internally processed and represented may be more crucial than exposure itself. This is in line with neurobiological accounts, as disrupted limbic–prefrontal connectivity—with diminished prefrontal constraint over amygdala reactivity and reduced hippocampal contextualisation—may lead to cascades of cognitive–affective dysfunction, i.e., a stress-related collapse of reflective functioning and dissociation [6, 23]. Our finding underscores the importance of trauma-related adaptation processes—particularly the capacity for reflective self- and other-understanding and the ability to regulate dissociative responses—in determining long-term outcomes. Such results lend further support to intervention approaches focused on enhancing mentalizing and reducing dissociative mechanisms in the aftermath of trauma (e.g., [75]).

While childhood and adulthood betrayal trauma showed comparable total effects on PD symptoms, their relationships with depressive symptoms diverged. Adulthood betrayal trauma demonstrated a stronger total association with depression than did childhood trauma. One plausible explanation is that adult trauma, being temporally closer to the present, remains more emotionally salient and less fully integrated, particularly when it occurs within ongoing relational contexts. Such experiences may interact with existing vulnerabilities—potentially shaped by earlier adversity—triggering depressive symptomatology among individuals whose coping and reflective capacities are already compromised [76]. Depression related to adult trauma may therefore reflect acute emotional dysregulation and unresolved. Moreover, adulthood betrayal trauma exhibited a weak indirect association with PD severity via dissociation and a comparatively stronger indirect association through hypomentalizing, while associations with depressive symptoms were relatively equally accounted for by both mechanisms. This asymmetry suggests that adult betrayal trauma may particularly compromise reflective capacities, which in turn sustain maladaptive personality functioning. The finding that adult trauma was more strongly linked to hypomentalizing aligns with evidence indicating that relational trauma interferes with the ability to understand and interpret mental states, thereby undermining affect regulation and interpersonal functioning [77]. Together, these results suggest that while both dissociation and hypomentalizing are important pathways within the observed associations between betrayal trauma and psychopathology, difficulties in mentalizing may play a more pivotal and enduring role, particularly in relation to personality pathology.

Given that individuals tend to develop more adaptive stress responses, enhanced affect regulation, and greater self-reflective capacity with age—even in the presence of relational adversity [6,11]—we hypothesised that trauma occurring in adulthood would be less strongly associated with impairments in overall personality functioning and more closely linked to symptomatic disorders than to enduring personality pathology. This hypothesis was only partially supported, as adulthood trauma showed comparable total effects on both depressive symptoms and PD severity. This finding suggests that trauma occurring in adulthood may not be limited to symptomatic outcomes but may also be associated with personality functioning. One possible explanation is that adulthood trauma may activate pre-existing vulnerabilities shaped by earlier developmental experiences or reflect cumulative exposure processes, rather than operating independently of personality structure. Moreover, the direct association of adulthood trauma on PD symptoms was negative and not statistically significant, whereas the indirect associations — via dissociation and hypomentalizing—were positive and of greater magnitude, yielding a positive total effect. This pattern further highlights the importance of psychological processing and suggests that the association between adulthood betrayal trauma and personality pathology becomes evident only when these mechanisms are taken into account. This result suggests that the principal source of psychological harm may lie not only in the traumatic event itself but also in how it is internally represented and processed, particularly through reflective engagement with trauma-related mental states. Hypomentalizing and dissociation thus appear to function as key psychological mechanisms linking adult relational trauma to enduring personality dysfunction, although longitudinal data are required to establish causal directionality. These findings are clinically and theoretically significant, given growing evidence that certain PDs—most notably borderline PD—are associated with both early and later interpersonal trauma. Such experiences often perpetuate cycles of relational dysfunction, including patterns of revictimisation in romantic or peer relationships [78, 79]. The moderate correlations observed between childhood and adulthood betrayal trauma in this study support this continuity: individuals exposed to early relational trauma appear more vulnerable to later betrayal experiences. This accords with theoretical models suggesting that adverse childhood experiences impair capacities necessary for forming and maintaining secure adult relationships [80]. In sum, including both childhood and adulthood betrayal trauma allowed us to examine their potentially distinct roles within a developmental framework. While childhood trauma may contribute to enduring vulnerabilities (e.g., in mentalizing or affect regulation), adulthood trauma may reflect more proximal stressors, including possible revictimization. The observed covariance between these variables is therefore consistent with cumulative risk models and does not undermine their conceptual distinction.

Our findings further indicate that mentalizing—more so than dissociation—served as the dominant pathway linking adulthood betrayal trauma to personality pathology, although other unmeasured factors undoubtedly contribute. This reinforces the conceptualisation of unmentalized traumatic experience as a core mechanism through which both early and later relational abuse and neglect give rise to enduring interpersonal and intrapsychic difficulties. Such difficulties are likely underpinned by compromised epistemic trust, attenuated social learning, and reduced access to the salutogenic potential of relationships, including the capacity to benefit from psychosocial and therapeutic interventions [7,81]. Despite increasing attention to early trauma, the distinct role and consequences of betrayal trauma in adulthood remain underexplored. Our results highlight the need for further research into the specific psychological processes through which adult relational trauma shapes personality functioning.

Across all models, mentalizing consistently exerted a stronger associations than dissociation, accounting for over half of the total indirect effects for both childhood and adulthood trauma in relation to both PD and depressive symptomatology. This supports the conceptualisation of mentalizing as a transdiagnostic mechanism implicated not only in personality pathology but also in symptomatic conditions such as depression. Although the relationship between mentalizing and depression has received growing empirical attention, findings remain mixed and appear to depend on measurement approach. For instance, Fischer-Kern et al. [82] identified reduced mentalizing capacity in patients with major depressive disorder (MDD) compared with healthy controls using clinician-rated assessments, whereas Taubner et al. [83] did not replicate this effect. In contrast, self-report measures of mentalizing have shown more robust associations with depressive symptoms—particularly with hypomentalization related the self [84] and in the context of early adversity [85, 39]. It is important to note that the mentalizing measure used in the present study primarily captures hypomentalizing, defined as uncertainty about mental states, particularly in relation to the self. However, hypomentalizing is a multidimensional construct that may take different forms. Recent research highlights that distinctions between self- and other-focused mentalizing, as well as between hypo- and hypermentalizing, may be especially important in shaping clinical presentations [32,86,87]. Accordingly, the present study does not capture other clinically relevant forms of mentalizing dysfunction, such as hypermentalizing or imbalances between self- and other-focused processing. These distinct patterns may be associated with different symptom profiles and mechanisms of psychopathology, and future research should aim to disentangle their specific contributions using more comprehensive and multi-method assessments.

Converging evidence also indicates that mentalizing constitutes a core mechanism of change across a range of psychotherapeutic modalities—not only in PD treatment but also in interventions for mood, trauma-related, and eating disorders, as well as adolescent self-harm [88, 89]. The current findings extend this literature by confirming the mediating role of mentalizing in depressive symptomatology, underscoring its critical role in the pathway from relational trauma to affective disturbance—mirroring its established function in the aetiology and maintenance of personality pathology [32,34,50].

### Limitations and future directions

Several limitations of the present study should be acknowledged. First, the sample comprised both clinical participants seeking psychological help and community participants with an interest in mental health. This recruitment strategy may have introduced selection bias, as such individuals could exhibit higher levels of psychological awareness, distress, or self-reflection than the general population, potentially inflating associations and limiting generalisability. At the same time, combining clinical and community participants may introduce heterogeneity related to differences in symptom severity and treatment history. To address this possibility, we conducted exploratory analyses examining whether recruitment source moderated the main associations. These analyses did not support moderation effects, suggesting that the observed patterns were relatively consistent across subsamples.

Second, the study relied on retrospective self-report measures of trauma, which are inherently susceptible to recall bias. Although prospective designs are often regarded as more robust, retrospective assessments remain valuable in both research and clinical contexts. Indeed, evidence shows that retrospective and prospective methods show only moderate agreement, each identifying largely distinct groups [90]. Nonetheless, retrospective reports may be more sensitive to subtle forms of maltreatment—such as emotional neglect, verbal abuse, or overprotection—that may be overlooked in prospective studies. Furthermore, our sample consisted entirely of participants from Poland**,** reflecting the study’s aim to contribute data from an Eastern European context. Prior work suggests that both the recall and the impact of traumatic events vary cross-culturally (e.g., [91]), and that the prevalence and expression of post-traumatic conditions are co-shaped by cultural and social factors—such as social acknowledgement and norms of disclosure, particularly for relational trauma [92]. Notably, recent population-based data from Poland indicate a comparatively high current prevalence of probable trauma-related disorders, mainly PTSD (≈19%, vs. ≈ 5–10% in other countries), with the highest conditional risk linked to childhood abuse and sexual assault [92]. Accordingly, while the findings are informative for understanding trauma-related processes in this specific cultural context, some caution is warranted when generalising beyond Poland. Another limitation concerns the uniform treatment of PD pathology: the analyses did not differentiate between specific PD diagnoses. Given that different PDs may arise from distinct developmental trajectories and mechanisms, future work should examine diagnosis-specific patterns. This also relates to a fourth limitation—the clinical heterogeneity of the sample—which may have obscured diagnostic or etiological nuances.

Another limitation involves the use of self-report measures to assess mentalizing. Mentalizing is a context-dependent capacity, dynamically expressed in interpersonal and affectively charged situations, particularly those involving attachment. Self-report tools lacking this relational or ecological context may therefore underestimate or mischaracterise individuals’ actual reflective functioning [28, 29]. Future studies should employ multimethod assessments that capture both self- and other-focused reflective capacities within attachment or trauma-relevant contexts, as suggested by [93], 2017). Moreover, we can’t exclude the possibility that shared method variance related to exclusive reliance on self-report instruments may inflate associations between trauma, dissociation, mentalizing, and symptom measures. [29] and Hawksdale et al. [28] have previously argued that different measures of mentalizing may address different relational contexts, with most measures assessing it in the context of a-specific relationships and only a smaller number of measures rating it in a more nuanced, and relational way. Mentalization is in fact a relational capacity that unfolds within the specific interpersonal context, and we cannot exclude that different measures (e.g., clinician-report or interview-based measures) could lead to different patterns of relationships between variables. Another limitation concerns the developmental scope of the study. Although trauma exposure may occur across multiple developmental periods, including adolescence, the present study focused on childhood and adulthood trauma as broad markers of early vulnerability and more proximal stress exposure. As a result, the potential specificity of trauma occurring during adolescence—a developmentally sensitive period for identity formation and personality functioning—was not examined. Future research should address this gap by more explicitly modelling adolescence as a distinct developmental window, ideally using designs that allow for more precise temporal assessment of trauma exposure.

Finally, the cross-sectional design and use of mediation analyses preclude causal inference. Although the proposed mediation pathways are grounded in theory and supported by prior empirical evidence, these analyses cannot determine the temporal ordering of variables [71]. Longitudinal and experimental designs are required to clarify causal mechanisms linking betrayal trauma, mentalizing, dissociation, and psychopathology. The cross-sectional nature of the data does not allow us to exclude the possibility of bidirectional relationships between key variables. In particular, while hypomentalizing may contribute to the development or maintenance of depressive symptoms, it is also plausible that current depressive states impair reflective functioning, leading to elevated reports of mentalizing deficits. Longitudinal or prospective designs are therefore necessary to disentangle whether hypomentalizing precedes psychopathology or emerges as a consequence of it. Additionally, the reliance on retrospective self-report measures of trauma introduces the possibility that current psychological states—particularly depressive symptoms—may influence the recall, interpretation, or reporting of past adverse experiences. Such mood-congruent recall biases may lead to either amplification or attenuation of reported trauma exposure, which should be taken into account when interpreting the observed associations.

### Conclusions and clinical implications

This study provides evidence that both mentalizing and dissociation play key roles in the relationship between betrayal trauma—whether in childhood or adulthood—and subsequent psychopathology. The findings highlight the importance of addressing these psychological processes in therapeutic work with traumatised individuals. Mentalizing, in particular, appears to represent a protective capacity that warrants careful assessment and targeted enhancement in clinical settings. Strengthening reflective capacities—especially trauma-related mentalizing—may help buffer the impact of adversity and prevent the development or maintenance of psychopathology. Empirical work has already demonstrated the value of interventions that explicitly focus on improving mentalizing in children exposed to trauma (e.g., [94]). The present results extend this evidence by confirming the central role of mentalizing in adult trauma survivors as well, underscoring its relevance across the lifespan. Moreover, these findings emphasise the need to recognise and address the impact of adult relational trauma on psychological functioning. More recently, a trauma-focused mentalization-based treatment approach has been developed to address the complex interplay of attachment disruptions, hypomentalizing and psychopathological outcomes related to what the authors address as attachment trauma [75], which can be considered as one of the forms that betrayal trauma can have [95]. Clinical interventions should not only focus on the consequences of early adversity but also attend to the cumulative and ongoing effects of later betrayals—particularly those that undermine epistemic trust and reflective engagement. The therapeutic relationship can be considered a significant relationship which can foster the development of epistemic trust and reflective functioning, however a history of betrayals by significant figures can undermine the capacity to rely and trust even mental health professionals: A careful and precise assessment of trauma history can be considered as a fundamental part of the case formulation. Therapeutic approaches that foster both reconnection with mental states and integration of fragmented experiences may therefore be essential for recovery and the restoration of psychological resilience.

## Supporting information

S1 Table Parallel Mediation Model: Comparison of Standardized Covariances: Satorra-Bentler (N = 209) vs. Bootstrap Methods (5000 resamples). *Note*. ^a^ Marital status: −1 = single and 1 = in romantic relationship.
(DOCX)

S2 Table Exploratory moderation analysis: Recruitment source as a moderator of trauma–mediator pathways. Note. Unstandardised (B) and standardised (β) coefficients are reported. Bootstrap estimates are based on 5,000 resamples with bias-corrected 95% confidence intervals. Recruitment source was coded as 0 = no treatment history, 1 = treatment history.
(DOCX)

S3 Text Inclusivity in Global Research Questionnaire. (PDF)
