# Supplementary material for: Betrayal trauma and adult mental health: The role of mentalizing and dissociation
Source: PLoS One. 2026 Jul 13;21(7):e0353662. doi: 10.1371/journal.pone.0353662 (PMC13362085; doi:10.1371/journal.pone.0353662)
Supplement: S1 Table — Note. a Marital status: −1 = single and 1 = in romantic relationship. (DOCX) [file pone.0353662.s001.docx]

**S1 Table**

*Parallel Mediation Model: Comparison of Standardized Covariances: Satorra-Bentler (N = 209) vs. Bootstrap Methods (5000 resamples)*

| Type | Relationship | β | *SE* | *z* | *p* | β | *SE* | *z* | *p* | *95% CI* |
| --- | --- | --- | --- | --- | --- | --- | --- | --- | --- | --- |
|  |  | Satorra-Bentler | Satorra-Bentler | Satorra-Bentler | Satorra-Bentler | Bootstrap | Bootstrap | Bootstrap | Bootstrap | Bootstrap |
| Between Outcomes | Depression ~~ Personality Disorders | 0.44 | 14.40 | 5.90 | <.001 | 0.44 | 14.24 | 5.96 | <.001 | [55.703, 111.589] |
| Between Mediators | Dissociation ~~ Mentalization Deficits | 0.39 | 5.93 | 5.64 | <.001 | 0.39 | 5.84 | 5.73 | <.001 | [21.453, 44.556] |
| Between Traumas | Childhood More-Betrayal ~~ Adulthood More-Betrayal | 0.53 | 0.57 | 5.45 | <.001 | 0.53 | 0.57 | 5.46 | <.001 | [2.033, 4.275] |
| Between Control Variables | Age ~~ Education | 0.30 | 0.69 | 4.04 | <.001 | 0.30 | 0.69 | 4.07 | <.001 | [1.432, 4.151] |
|  | Age ~~ Marital Status ^a^ | 0.12 | 0.92 | 1.81 | .070 | 0.12 | 0.92 | 1.80 | .071 | [-0.19, 3.465] |
|  | Education ~~ Marital Status ^a^ | 0.25 | 0.14 | 3.68 | <.001 | 0.25 | 0.14 | 3.75 | <.001 | [0.247, 0.801] |
| Control Variables with Trauma | Age ~~ Childhood trauma | -0.06 | 1.49 | -0.91 | .363 | -0.06 | 1.48 | -0.92 | .359 | [-4.168, 1.604] |
|  | Education ~~ Childhood trauma | -0.21 | 0.23 | -2.94 | .003 | -0.21 | 0.23 | -2.95 | .003 | [-1.138, -0.23] |
|  | Marital Status ^a^ ~~ Childhood trauma | -0.16 | 0.33 | -2.36 | .018 | -0.16 | 0.33 | -2.37 | .017 | [-1.425, -0.149] |
|  | Age ~~ Adulthood trauma | 0.11 | 1.26 | 1.46 | .143 | 0.11 | 1.26 | 1.46 | .144 | [-0.593, 4.362] |
|  | Education ~~ Adulthood trauma | -0.22 | 0.17 | -3.13 | .001 | -0.22 | 0.18 | -3.10 | .001 | [-0.881, -0.188] |
|  | Marital Status ^a^ ~~ Adulthood trauma | -0.10 | 0.24 | -1.53 | .125 | -0.10 | 0.23 | -1.54 | .123 | [-0.82, 0.094] |
| Control Variables with Mediators | Age ~~ Dissociation | -0.32 | 11.24 | -4.83 | <.001 | -0.32 | 11.22 | -4.84 | <.001 | [-75.663, -31.926] |
|  | Education ~~ Dissociation | -0.37 | 1.83 | -5.22 | <.001 | -0.37 | 1.81 | -5.28 | <.001 | [-13.041, -5.853] |
|  | Marital Status ^a^ ~~ Dissociation | -0.21 | 2.72 | -2.92 | .003 | -0.21 | 2.74 | -2.90 | .003 | [-13.25, -2.556] |
|  | Age ~~ Mentalization | -0.06 | 2.35 | -0.82 | .409 | -0.06 | 2.35 | -0.82 | .409 | [-6.397, 2.866] |
|  | Education ~~ Mentalization | -0.22 | 0.33 | -3.20 | .001 | -0.22 | 0.32 | -3.22 | .001 | [-1.656, -0.383] |
|  | Marital Status ^a^ ~~ Mentalization | -0.27 | 0.50 | -3.76 | <.001 | -0.27 | 0.50 | -3.76 | <.001 | [-2.847, -0.869] |
| Control Variables with Outcomes | Age ~~ Depression | -0.18 | 2.97 | -2.43 | .014 | -0.18 | 2.94 | -2.46 | .014 | [-12.838, -1.291] |
|  | Education ~~ Depression | -0.25 | 0.42 | -3.67 | <.001 | -0.25 | 0.41 | -3.73 | <.001 | [-2.304, -0.696] |
|  | Marital Status ^a^ ~~ Depression | -0.01 | 0.58 | -0.22 | .827 | -0.01 | 0.58 | -0.22 | .826 | [-1.285, 1.007] |
|  | Age ~~ Personality disorders | -0.04 | 19.40 | -0.60 | .550 | -0.04 | 19.27 | -0.60 | .547 | [-48.413, 27.512] |
|  | Education ~~ Personality disorders | -0.17 | 3.23 | -2.33 | .020 | -0.17 | 3.24 | -2.32 | .020 | [-13.832, -0.941] |
|  | Marital Status ^a^ ~~ Personality disorders | -0.11 | 4.40 | -1.68 | .093 | -0.11 | 4.41 | -1.68 | .093 | [-15.955, 1.392] |

*Note*. ^a^ Marital status: -1 = single and 1 = in romantic relationship.
