# Supplementary material for: Betrayal trauma and adult mental health: The role of mentalizing and dissociation
Source: PLoS One. 2026 Jul 13;21(7):e0353662. doi: 10.1371/journal.pone.0353662 (PMC13362085; doi:10.1371/journal.pone.0353662)
Supplement: S2 Table — Note. Unstandardised (B) and standardised (β) coefficients are reported. Bootstrap estimates are based on 5,000 resamples with bias-corrected 95% confidence intervals. Recruitment source was coded as 0 = no treatment history, 1 = treatment history. (DOCX) [file pone.0353662.s002.docx]

Table S2.

Exploratory moderation analysis: Recruitment source as a moderator of trauma–mediator pathways

| Predictor | Outcome | *B* | β | *SE* | *z* | *p* | 95% *CI* |
| --- | --- | --- | --- | --- | --- | --- | --- |
| Childhood trauma × recruitment source → Dissociation | Dissociation | 6.06 | 0.09 | 6.90 | 0.88 | .380 | [-7.714, 19.406] |
| Adulthood trauma × recruitment source → Dissociation | Dissociation | -0.26 | -0.00 | 10.19 | -0.03 | .979 | [-21.127, 19.245] |
| Childhood trauma × recruitment source → Mentalization | Mentalization | 1.88 | 0.15 | 1.20 | 1.56 | .119 | [-0.526, 4.182] |
| Adulthood trauma × recruitment source → Mentalization | Mentalization | -0.27 | -0.02 | 1.82 | -0.15 | .881 | [-3.977, 3.238] |

*Note.* Unstandardised (B) and standardised (β) coefficients are reported. Bootstrap estimates are based on 5,000 resamples with bias-corrected 95% confidence intervals. Recruitment source was coded as 0 = no treatment history, 1 = treatment history.
