# Supplementary material for: Betrayal trauma and adult mental health: The role of mentalizing and dissociation
Source: PLoS One. 2026 Jul 13;21(7):e0353662. doi: 10.1371/journal.pone.0353662 (PMC13362085; doi:10.1371/journal.pone.0353662)
Supplement: Attachment — Submitted filename: 02_PONE_Response Letter.pdf [file pone.0353662.s005.pdf]

PONE-D-25-62663
Betrayal Trauma and Adult Mental Health: The Mediating Role of Mentalizing and Dissociation
PLOS One

We would like to sincerely thank the Editor for the careful evaluation of our manuscript and for recognizing the importance and timeliness of the topic. We appreciate the constructive feedback and the opportunity to revise and resubmit our work. Below, we provide a detailed, point-by-point response to all concerns raised and describe the revisions made to the manuscript accordingly.

Monika O. Jańczak, on behalf of all co-authors

Journal Requirements:
1. Please ensure that your manuscript meets PLOS ONE's style requirements, including those for file naming. The PLOS ONE style templates can be found at ..

We have revised the manuscript to ensure full compliance with PLOS ONE style and formatting requirements, including file naming conventions. All figures have been prepared and submitted in accordance with the journal's technical specifications.

2. Please include a complete copy of PLOS' questionnaire on inclusivity in global research in your revised manuscript. Our policy for research in this area aims to improve transparency in the reporting of research performed outside of researchers' own country or community. The policy applies to researchers who have travelled to a different country to conduct research, research with Indigenous populations or their lands, and research on cultural artefacts. The questionnaire can also be requested at the journal's discretion for any other submissions, even if these conditions are not met. Please find more information on the policy and a link to download a blank copy of the questionnaire here: https://journals.plos.org/plosone/s/best-practices-in-research-reporting. Please upload a completed version of your questionnaire as Supporting Information when you resubmit your manuscript.

We have included a completed copy of PLOS' questionnaire on inclusivity in global research as Supporting Information with the revised manuscript, in accordance with the journal's policy.

3. Thank you for stating the following in the Acknowledgments Section of your manuscript: "This research was funded by the Adam Mickiewicz University Program ID-UB "Initiative of Excellence – AMU Research University," project no. 037/02/POB5/0027, awarded to MOJ (Monika Olga Jańczak). Funder website: https://amu.edu.pl Manuscript Click here to access/download;Manuscript;02_BODY_TraumaRelated Psychopathology.docx The funder had no role in study design, data collection and analysis, decision to publish, or preparation of the manuscript." We note that you have provided funding information that is not currently declared in your Funding Statement. However, funding information should not appear in the Acknowledgments section or other areas of your manuscript. We will only publish funding information present in the Funding Statement section of the online submission form. Please remove any funding-related text from the manuscript and let us know how you would like to update your Funding Statement. Currently, your Funding Statement reads as follows: "This research was funded by the Adam Mickiewicz University Program ID-UB "Initiative of Excellence – AMU Research University," project no. 037/02/POB5/0027, awarded to MOJ (Monika Olga Jańczak). Funder website: https://amu.edu.pl The funder had no role in study design, data collection and analysis, decision to publish, or preparation of the manuscript." Please include your amended statements within your cover letter; we will change the online submission form on your behalf.

Thank you for the clarification. We have removed all funding-related information from the manuscript.

We confirm that the Funding Statement should be as follows ::

“This research was funded by the Adam Mickiewicz University Program ID-UB ‘Initiative of Excellence – AMU Research University,’ project no. 037/02/POB5/0027, awarded to MOJ (Monika Olga Jańczak). Funder website: https://uczelniabadawcza.amu.edu.pl. The funder provided support in the form of salaries for author [MOJ], but did not have any additional role in the study design, data collection and analysis, decision to publish, or preparation of the manuscript. The specific roles of these authors are articulated in the ‘author contributions’ section.”

4. We note that the grant information you provided in the ‘Funding Information’ and ‘Financial Disclosure’ sections do not match.
When you resubmit, please ensure that you provide the correct grant numbers for the awards you received for your study in the ‘Funding Information’ section.

Thank you for noting this. We have reviewed and corrected the grant information to ensure that the ‘Funding Information’ section matches the ‘Financial Disclosure’ section.

5. Thank you for stating the following financial disclosure:
“This research was funded by the Adam Mickiewicz University Program ID-UB “Initiative of Excellence – AMU Research University,” project no. 037/02/POB5/0027, awarded to MOJ (Monika Olga Jańczak). Funder website: https://amu.edu.pl The funder had no role in study design, data collection and analysis, decision to publish, or preparation of the manuscript.”
We note that one or more of the authors is affiliated with the funding organization, indicating the funder may have had some role in the design, data collection, analysis or preparation of your manuscript for publication; in other words, the funder played an indirect role through the participation of the co-authors. If the funding organization did not play a role in the study design, data collection and analysis, decision to publish, or preparation of the manuscript and only provided financial support in the form of authors' salaries and/or research materials, please do the following:
1. Review your statements relating to the author contributions, and ensure you have specifically and accurately indicated the role(s) that these authors had in your study. These amendments should be made in the online form.
2. Confirm in your cover letter that you agree with the following statement, and we will change the online submission form on your behalf:
“The funder provided support in the form of salaries for authors [insert relevant initials], but did not have any additional role in the study design, data collection and analysis, decision to publish, or preparation of the manuscript. The specific roles of these authors are articulated in the ‘author contributions’ section.

With regard to the funding disclosure, we would like to clarify that the grant provided by the Adam Mickiewicz University Program ID-UB covered research-related costs only (e.g., participant compensation and recruitment expenses). No authors received salaries or personal remuneration from this funding source in relation to this project. The authors’ affiliation with the funding institution reflects standard academic employment and is not related to the funding of this project. The funder had no role in the study design, data collection and analysis, decision to publish, or preparation of the manuscript. We therefore confirm the Funding Statement as follows:

“This research was funded by the Adam Mickiewicz University Program ID-UB ‘Initiative of Excellence – AMU Research University,’ project no. 037/02/POB5/0027, awarded to MOJ (Monika Olga Jańczak). Funder website: https://uczelniabadawcza.amu.edu.pl. The funder provided support in the form of salaries for author [MOJ], but did not have any additional role in the study design, data collection and analysis, decision to publish, or preparation of the manuscript. The specific roles of these authors are articulated in the ‘author contributions’ section.”

6. Please include captions for your Supporting Information files at the end of your manuscript, and update any in-text citations to match accordingly. Please see our Supporting Information guidelines for more information: http://journals.plos.org/plosone/s/supporting-information.

We have added captions for all Supporting Information files at the end of the manuscript and updated the in-text citations accordingly to ensure consistency with PLOS ONE guidelines.

Additional Editor Comments:

The topic of this study is interesting. It is a timely and important investigation. However, I do have several concerns and therefore would like to invite you to revise and resubmit before I send it out for peer review.

We sincerely appreciate the Editor's positive evaluation of the relevance and timeliness of our study. We are grateful for the opportunity to revise and resubmit the manuscript and have carefully addressed all concerns raised.

First of all, the relationship between betrayal trauma and dissociation / dissociative symptoms/features has been increasingly studied in the recent literature, but the authors failed to review the recent and most relevant literature. I would expect a more detailed, updated literature review, especially from the cross-cultural perspective, given the sample of your study.

We thank the Editor for this important comment and fully agree that the initial version of the manuscript did not sufficiently reflect the most recent and relevant literature on betrayal trauma and dissociation. We acknowledge this as a significant oversight. In response, we have substantially revised the Introduction by adding a new paragraph that integrates recent empirical findings on the association between betrayal trauma and dissociation, including studies conducted in both English-speaking countries and culturally diverse contexts.

In addition, the cultural relevance of the current study has been more clearly articulated throughout the manuscript. Specifically, we have strengthened the cross-cultural framing in the Abstract, clarified this focus in the Aims of the study, and further elaborated on the contribution of data from an underrepresented Eastern European context in the Discussion.

Second, it is not acceptable and scientifically convincing enough to use cross-sectional data to explore or reveal mediating effects. Please reconsider what your data can and cannot tell.

Thank you for this important methodological comment. We fully agree that cross-sectional data do not allow for causal inference or for establishing temporal mediation mechanisms. In response, we have revised the manuscript to more clearly reflect what our data can support. Specifically, we have changed the title and removed the term "mediating" from all section headings, figure titles, and table titles, in order to avoid any implication that the identified pathways represent causal or developmental mechanisms. We have retained the use of mediation terminology in the statistical analysis section only, where it is explicitly framed as statistical mediation within a path-analytic framework, not as evidence of causal or temporal processes. Furthermore, references to mediation in the Aims, Abstract, and Discussion have been either removed or carefully reformulated to emphasize indirect associations and model-based pathways, with explicit clarification that these analyses are cross-sectional and exploratory in nature. We believe that these revisions appropriately align the interpretation of our findings with the limitations of the study design, while preserving a transparent and accurate description of the statistical methods employed.
