# Supplementary material for: Betrayal trauma and adult mental health: The role of mentalizing and dissociation
Source: PLoS One. 2026 Jul 13;21(7):e0353662. doi: 10.1371/journal.pone.0353662 (PMC13362085; doi:10.1371/journal.pone.0353662)
Supplement: Attachment — Submitted filename: PONE_Response to Reviewers.pdf [file pone.0353662.s006.pdf]

PONE-D-25-62663

Betrayal Trauma and Adult Mental Health: The Mediating Role of Mentalizing and Dissociation

PLOS One

We sincerely thank the Reviewer for the careful and thorough evaluation of our manuscript and for acknowledging the relevance and timeliness of the topic. We are grateful for the constructive comments and the opportunity to revise and resubmit our work. Below, we provide a detailed, point-by-point response to all issues raised and outline the corresponding revisions made to the manuscript.

Monika O. Jańczak, on behalf of all co-authors

Reviewer's comments:

#1: Abstract

1. There is a slight inconsistency between the background and the objective. The background discusses the psychological mechanisms of trauma, while the objective focuses on the relationship between dissociation and ineffective mental mapping. It is recommended to revise this section to ensure consistency with the findings.

We have revised the Abstract to make the link between the background and the study aims clearer and to ensure consistency with the Reviewer's further comments regarding cultural differences.

2. Regarding the participants, please briefly describe the source and duration of recruitment.

The source and duration of participant recruitment have now been briefly described in the Abstract.

3. Because of the path analysis, please include each hypothetical path in the objective section.

Following the Reviewer's suggestion, we have revised the Objective section to explicitly include the hypothesized paths tested in the path analysis.
As revisions to the Abstract resulted in increased length, we condensed the Results section to improve clarity and conciseness.

Introduction

1. Since the researchers did not intend to measure resilience, it is recommended to change "Mentalizing and dissociation as resilience and risk factors following trauma" to "Mentalizing and dissociation as protective and risk factors following trauma."

We have revised the terminology throughout the manuscript to avoid implying that resilience was directly assessed. Following the Reviewer's suggestion, we consistently use the terms "protective factors" and "risk factors" instead.

2. The logic of the validation framework is unclear. It fails to infer why childhood betrayal trauma affects symptoms of personality disorders and depression, while adult betrayal trauma affects depressive symptoms. Which type of personality disorder does PD refer to? These should be explained in detail.

We thank the Reviewer for highlighting the need to clarify the logic of the validation framework. In the revised manuscript, we have clarified this logic by more explicitly highlighting the arguments already presented in the Introduction. In particular, drawing on a developmental perspective on the timing of relational trauma, we emphasize that betrayal trauma occurring earlier in life is theorized to interfere with the formation of core self–other representations, attachment patterns, and regulatory capacities, thereby exerting more pervasive effects on personality functioning as well as mood-related symptoms. In contrast, betrayal trauma occurring in adulthood is assumed to impact an already consolidated personality structure, leading primarily to affective and symptomatic manifestations, such as depressive symptoms. We also adjusted the study aims accordingly.

We also clarify thorough the text that PD refers to the level of personality functioning within a dimensional PD model (as in ICD-11 and Alternative Model of PD in DSM-5), rather than to specific type of personality disorder. We also added a relevant line in the introduction.

3. If a comparison of cultural differences is to be made, this study did not include participants from other cultures for comparison, so it is impossible to understand why cultural differences exist.

We agree with the Reviewer that the present study does not allow for direct comparisons of cultural differences, as it does not include participants from multiple cultural contexts. In the revised manuscript, we have clarified this point and revised the text to avoid implying cross-cultural comparisons. We now frame the study as contributing data from an underrepresented population rather than making claims about cultural differences.

Overall, this topic is interesting. However, I could not fully understand about the rationale of the hypothesized paths in the present study.

We thank the Reviewer for this comment. We hope that the revisions made to the manuscript have clarified the rationale underlying the hypothesized paths.
