# Supplementary material for: Betrayal trauma and adult mental health: The role of mentalizing and dissociation
Source: PLoS One. 2026 Jul 13;21(7):e0353662. doi: 10.1371/journal.pone.0353662 (PMC13362085; doi:10.1371/journal.pone.0353662)
Supplement: Attachment — Submitted filename: REV2_Response to Reviewers PLOS.pdf [file pone.0353662.s007.pdf]

PONE-D-25-62663

Betrayal Trauma and Adult Mental Health: The Mediating Role of Mentalizing and Dissociation

PLOS One

We sincerely thank the Reviewers for the careful and thorough evaluation of our manuscript and for acknowledging the relevance and timeliness of the topic. We are grateful for the constructive comments and the opportunity to revise and resubmit our work. Below, we provide a detailed, point-by-point response to all issues raised and outline the corresponding revisions made to the manuscript. All changes have been implemented in the revised version and are visible using tracked changes. Where feasible, we have included excerpts of the revisions directly in our responses; however, longer or more dispersed changes are indicated only in the tracked manuscript.

Monika O. Jańczak, on behalf of all co-authors

Reviewer's comments

Reviewer #1: Thanks for considering my comments and suggestions. The revised manuscript is more comprehensive. Only one suggestion is to consider the necessarity of "neurobiological mechanisms of trauma". Actually, the present study did not collect the biological data, such as neuroimage or biological markers. Please to focus on the psychological mechanism of trauma.

Thank you for this helpful suggestion. In response to this comment and the comment of Reviewer 2, we have substantially shortened the section on neurobiological mechanisms and retained only a minimal reference to neurodevelopmental evidence to support the possible distinct consequences between childhood and adulthood trauma.

Reviewer #2: The authors may consider further strengthening the conceptual clarity of the manuscript by tightening the alignment between the theoretical framework (developmental psychopathology and betrayal trauma theory) and the specific statistical model tested. While the cascade model is well described, the justification for positioning dissociation and mentalizing strictly as parallel (rather than sequential or interacting) mediators could be elaborated further.

Thank you for this comment. In response, we have revised the Aims section to more explicitly align the statistical model with the theoretical framework discussed in the Introduction regarding the relationship between mentalizing and dissociation.

It is now at follows:

Aims:

It was further hypothesised that higher levels of betrayal trauma would correspond with greater ineffective mentalizing and higher dissociation, particularly for childhood trauma. Ineffective mentalizing and dissociation were expected to show positive associations with both depression and PD severity. Finally, consistent with the developmental psychopathology framework proposed by Bateman and Fonagy (2019), it was anticipated that ineffective mentalizing would be positively correlated with dissociation. Given the mixed and bidirectional findings in prior research, and the lack of consistent evidence supporting a unidirectional pathway, the present study modelled dissociation and mentalizing as parallel mediators, capturing their potentially independent yet interrelated contributions to trauma-related psychopathology.

The authors may consider clarifying the conceptual distinction between "ineffective mentalizing" and "hypomentalizing," particularly given that only the RFQ_U subscale was used (pp. 22–23). A brief

rationale for excluding RFQ_C and a discussion of the implications for construct coverage would strengthen interpretability.

We agree that ineffective mentalizing is a multidimensional construct that may manifest in different forms, including both hypomentalizing (uncertainty about mental states) and hypermentalizing (overinterpretation or unwarranted certainty). Moreover, recent research suggests that distinctions such as self–other imbalance and hyper- versus hypomentalizing may differentially shape clinical presentations and psychopathology. Therefore, it is important to explicitly specify which facet of mentalizing is captured in the present study.

In this study, we focused on the RFQ_U subscale as an index of hypomentalizing, reflecting uncertainty and a reduced capacity to infer mental states. This decision was guided both by theoretical and methodological considerations. From a theoretical perspective, impairments in self-focused mentalizing appear to play a particularly central role in psychopathology, especially in relation to affect regulation and vulnerability to depressive and personality pathology (Fonagy, Bateman, 2019; Jańczak et al., 2025). From a methodological standpoint, prior validation studies indicate that RFQ_U demonstrates more robust psychometric properties and clearer interpretability compared to RFQ_C, which has been shown to be more difficult to interpret and may capture heterogeneous processes, including both adaptive certainty and maladaptive overconfidence (Müller et al., 2020; Woźniak-Prus et al., 2022).

At the same time, we acknowledge that relying exclusively on RFQ_U limits the scope of mentalizing assessment, as it does not capture hypermentalizing or more nuanced self–other distinctions. This constraint may have reduced our ability to detect differential patterns associated with specific forms of mentalizing dysfunction. We have now clarified this issue in the Methods section and elaborated on its implications in the Discussion.

Methods

Reflective Functioning Questionnaire (RFQ-8; Fonagy et al., 2016). *The RFQ-8 is an eight-item self-report measure of mentalizing. Each item is rated on a seven-point Likert scale (1 = strongly disagree to 7 = strongly agree). The questionnaire includes two subscales: certainty (RFQ_C) and uncertainty (RFQ_U) about mental states. In the present study, we focused on the RFQ_U subscale, which primarily captures hypomentalizing, defined as uncertainty about mental states, particularly in relation to the self. This decision was based on prior psychometric evidence indicating that RFQ_U demonstrates more robust properties and clearer interpretability compared to RFQ_C, which has been questioned as a valid indicator of ineffective mentalizing (e.g., Müller et al., 2020; Woźniak-Prus et al., 2022). In addition, focusing exclusively on RFQ_U avoids methodological issues related to double-scoring and the mathematical dependence between the two subscales, while remaining consistent with more recent unidimensional approaches to RFQ scoring (Müller et al., 2020; Woźniak-Prus et al., 2022). This approach has also been adopted in recent studies using the RFQ (e.g., Bateman et al., 2023). The validated Polish version (Woźniak-Prus et al., 2022) was used and demonstrated acceptable internal consistency in the present sample (Cronbach's $\alpha = 0.79$).*

Discussion:
It is important to note that the mentalizing measure used in the present study primarily captures hypomentalizing, defined as uncertainty about mental states, particularly in relation to the self. However, ineffective mentalizing is a multidimensional construct that may take different forms. Recent research highlights that distinctions between self- and other-focused mentalizing, as well as between hypo- and hypermentalizing, may be especially important in shaping clinical presentations (Barnby et al., 2025; Jańczak et al., 2025; Jańczak et. al. 2026). Accordingly, the present study does not capture other clinically relevant forms of mentalizing dysfunction, such as hypermentalizing or imbalances between self- and other-focused processing. These distinct patterns may be associated with different symptom profiles and mechanisms of psychopathology, and future research should aim to disentangle

their specific contributions using more comprehensive and multi-method assessments.

The authors may consider more explicitly articulating the theoretical rationale for including both childhood and adulthood betrayal trauma in the same model, especially given their moderate covariance (Table 1; pp. 27–28). Clarifying whether adulthood trauma is conceptualized as independent exposure, continuation, or potential revictimization would improve theoretical precision.

We agree that clarifying the conceptual rationale for including both childhood and adulthood betrayal trauma in the same model strengthens the theoretical precision of the study.

In the present framework, childhood and adulthood betrayal trauma were conceptualised as developmentally distinct but potentially related exposures. Childhood trauma was treated as an early vulnerability factor that may shape the development of mentalizing capacities and dissociative tendencies, thereby exerting long-term effects on personality pathology and emotional functioning. In contrast, adulthood trauma was conceptualised as a more proximal stressor, reflecting either independent exposure or, in some cases, potential revictimization linked to earlier vulnerabilities.

The inclusion of both indices in a single model was intended to disentangle their unique and shared contributions to psychopathology, while accounting for their natural co-occurrence. The observed covariance between childhood and adulthood trauma is consistent with prior research on revictimization and cumulative adversity and was therefore modelled explicitly rather than treated as a confound.

We have now clarified this rationale in the Introduction and Discussion sections of the manuscript.

The authors may consider streamlining the neurobiological section (pp. 14–15) to focus more directly on mechanisms that are empirically linked to dissociation and mentalizing. While comprehensive, some neuroimaging findings appear somewhat distal to the specific mediation model tested.

Thank you for this helpful suggestion. In response to this comment and the comment of Reviewer 1, we have substantially shortened the section on neurobiological mechanisms and retained only a minimal reference to neurodevelopmental evidence to support the possible distinct consequences between childhood and adulthood trauma.

The authors may consider more clearly differentiating between betrayal trauma and broader interpersonal trauma constructs early in the introduction. Although betrayal trauma theory is referenced, a sharper operational definition would help readers understand how it uniquely informs the hypotheses.

In line with this suggestion, we have revised the Introduction to provide a more explicit operational definition of betrayal trauma.

Introduction:

Complementing this, betrayal trauma theory differentiates adverse experiences by the degree of betrayal involved: maltreatment perpetrated by a trusted other within a relationship of attachment or dependency constitutes a specific subtype of interpersonal trauma, distinct from harm inflicted by non-significant others (Goldberg & Freyd, 2006; Freyd, 1994). Such experiences may entail unique adaptational demands.

The authors may consider clarifying the logic underlying the hypothesis that adulthood trauma would primarily relate to depression but not PD severity (pp. 18–19), particularly given that the results

showed comparable total effects on PD severity. A more cautious phrasing of the hypothesis or a clearer developmental argument may be warranted.

We thank the Reviewer for this helpful comment. In response, we have expanded the rationale in the Aims section to further clarify the developmental logic underlying our hypothesis. We have also revised the Discussion to more explicitly acknowledge that this hypothesis was only partially supported, and to more clearly address the discrepancy between the expected and observed patterns, which was not sufficiently highlighted in the previous version of the manuscript.

Aims:

In contrast, given that personality structure is largely consolidated by adulthood, trauma occurring later in life may function more as a proximal stressor linked to depressive symptoms than as a factor shaping enduring personality pathology.

Discussion:

This hypothesis was only partially supported, as adulthood trauma showed comparable total effects on both depressive symptoms and PD severity. This finding suggests that trauma occurring in adulthood may not be limited to symptomatic outcomes but may also be associated with personality functioning. One possible explanation is that adulthood trauma may activate pre-existing vulnerabilities shaped by earlier developmental experiences or reflect cumulative exposure processes, rather than operating independently of personality structure.

The authors may consider providing more detail regarding the Polish translation of the BBTS (p. 22). Specifically, information about translation procedures (e.g., back-translation, pilot testing) would strengthen confidence in measurement validity.

We agree that providing additional detail on the translation procedure strengthens confidence in the validity of the measure. We have now expanded the description of the Polish adaptation of the BBTS in the Methods section. A more detailed description of the adaptation and validation procedure is provided in a separate manuscript currently in preparation (Sienski & Jańczak, in preparation), which we now cite in the text.

Methods:

A Polish translation of the BBTS was developed using a multi-step procedure. Three independent forward translations were prepared by experienced clinicians with advanced proficiency in English, followed by expert panel reconciliation. The reconciled version was then back-translated into English by an independent translator experienced in academic translation and compared with the original instrument to ensure semantic and conceptual equivalence. The final version was reviewed in consultation with the original author of the instrument (J. Freyd). A more detailed description of the adaptation and validation process is provided elsewhere (Sienski & Jańczak, in preparation).

The authors may consider reporting the proportion and pattern of missing data prior to missForest imputation (p. 24). Transparency regarding the percentage of missingness per variable would enhance methodological clarity.

We thank the reviewer for this helpful comment. We have now clarified the proportion and pattern of missing data in the Methods section. The overall proportion of missing data was low (1.10%), and no systematic pattern of missingness was observed.

The authors may consider clarifying whether recruitment source (clinical vs. community; p. 21) was tested as a moderator or covariate. Given the mixed sample, subgroup differences could meaningfully influence associations.

We appreciate this important comment. The present study was designed as a dimensional, transdiagnostic investigation rather than a categorical comparison of recruitment groups. Accordingly, participants were recruited from both clinical and community settings to maximise variability in trauma exposure, personality pathology, and depressive symptoms, thereby enabling the examination of mechanisms across a broader severity continuum. This dimensional approach is consistent with contemporary frameworks of psychopathology and constitutes a strength of the study, as it allows for testing associations across naturally occurring variability rather than within restricted subgroups.

At the same time, we acknowledge that recruitment source may represent a potential source of heterogeneity, and we agree that such differences should be taken into account to improve the interpretability of the findings. Therefore, in response to the reviewer's comment and to strengthen the robustness of our conclusions, we conducted additional exploratory analyses to examine whether recruitment source alters the structural relations of interest.

To address potential heterogeneity related to recruitment source, we conducted an exploratory moderation analysis testing whether recruitment source (clinical vs. community sample) moderated the associations between betrayal trauma and the mediators. Interaction effects were not supported by bootstrap estimates. Specifically, the interaction between childhood trauma and recruitment source predicting dissociation was non-significant ($B = 6.06$, $\beta = .09$, $p = .380$, 95% CI [−7.714, 19.406]), as was the corresponding interaction for adulthood trauma ($B = -0.26$, $\beta = -.00$, $p = .979$, 95% CI [−21.127, 19.245]). Similarly, no robust moderation effects were observed for mentalization pathways (childhood trauma: $B = 1.88$, $\beta = .15$, $p = .119$, 95% CI [−0.526, 4.182]; adulthood trauma: $B = -0.27$, $\beta = -.02$, $p = .881$, 95% CI [−3.977, 3.238]). Moreover, the overall fit of the moderation model was poor, $\chi^2(22) = 412.56$, $p < .001$, CFI = .689, TLI = .066, RMSEA = .291, SRMR = .173, indicating that the inclusion of interaction terms substantially reduced model fit.

Taken together, these findings suggest that recruitment source does not meaningfully influence the associations between betrayal trauma and the proposed mediators, and that the observed pathways operate similarly across participants recruited from clinical and community settings. This supports the validity of modelling these processes dimensionally across a mixed sample. Given the absence of moderation effects and the already parameter-rich nature of the primary model, recruitment source was not included in the final path model to preserve parsimony and interpretability.

We have now clarified this rationale in the manuscript and report the results of the exploratory moderation analysis in Table S2 in the Supplementary Materials. We have clarified this issue across the manuscript by adding brief statements in the Aims (highlighting the dimensional, transdiagnostic rationale), Statistical Analysis (describing the exploratory examination of recruitment source), Results (summarising the absence of moderation effects), and Limitations (acknowledging potential heterogeneity while noting the robustness of findings across subsamples).

The authors may consider justifying the exclusion of adolescence trauma indices from the BBTS (p. 22), especially as adolescence represents a developmentally sensitive period relevant to personality functioning.

We thank the reviewer for this important comment. We agree that adolescence represents a developmentally sensitive period relevant to personality functioning. Nevertheless, in the present study, we focused on childhood and adulthood betrayal trauma to capture early developmental vulnerability and more proximal stress exposure within a parsimonious analytical framework. The

exclusion of adolescence was motivated by the focused scope of the research question (i.e., the role of childhood versus adulthood trauma), as well as by the need to limit model complexity and considerations related to retrospective measurement. Specifically, given that trauma exposure was assessed retrospectively, distinctions between developmental periods, such as adolescence and early adulthood, may be less reliably reported compared to broader contrasts (e.g., childhood vs. adulthood). Moreover, to reduce potential recall bias, the sample was restricted to younger and middle-aged adults; however, even within this age range, precise differentiation of trauma timing across adolescence may remain less robust.

However, we acknowledge that adolescence may represent a distinct developmental window with potentially unique implications for personality functioning. This limitation has now been explicitly noted in the manuscript, and future research should examine adolescence trauma more directly, ideally using longitudinal or prospective designs.

Limitations:
Another limitation concerns the developmental scope of the study. Although trauma exposure may occur across multiple developmental periods, including adolescence, the present study focused on childhood and adulthood trauma as broad markers of early vulnerability and more proximal stress exposure. As a result, the potential specificity of trauma occurring during adolescence—a developmentally sensitive period for identity formation and personality functioning—was not examined. Future research should address this gap by more explicitly modelling adolescence as a distinct developmental window, ideally using designs that allow for more precise temporal assessment of trauma exposure.

Statistical Analysis and Interpretation
The authors may consider presenting unstandardized coefficients alongside standardized betas in supplementary materials to facilitate replication and meta-analytic integration.

We have added unstandardized coefficients alongside standardized estimates (β) and now report them directly in the main results table (now Table 2) to enhance transparency of the analyses.

The authors may consider elaborating on the suppression effect observed in the adulthood trauma–PD pathway (pp. 30–31). Although described as a statistical artefact, a more nuanced theoretical reflection on possible interpretative scenarios would add depth.

Thank you for this thoughtful suggestion. We agree that the observed pattern in the adulthood trauma–PD pathway may invite further theoretical reflection. In the previous version of the manuscript, we initially attempted to provide such a theoretical interpretation in the Discussion. However, we realised that this was not fully consistent with our description of the same pattern in the Results section, where it was characterised as a statistical artefact. This inconsistency was unintentional and reflected our effort to balance methodological caution with theoretical interpretation.

Following the Reviewer's comment, we revisited this issue and carefully reconsidered the most appropriate way to present this finding. While we did not fully adopt the suggested approach of elaborating a detailed theoretical interpretation of suppression, we believe that the revised version provides a more methodologically sound and internally consistent account of the findings. In particular, the Reviewer's comment helped us identify this inconsistency and prompted us to clarify our interpretation.

Given that the direct effect was very small and non-significant, we concluded that there are insufficient grounds to interpret this pattern as a meaningful suppression effect. Instead, it is more appropriately understood as a statistical artefact arising from the combination of a near-zero direct effect and substantial indirect effects. This pattern is more plausibly explained by a proportional

artefact and is therefore more consistent with both statistical principles and theoretical expectations. Interpreting this pattern as a meaningful suppression effect would imply a counterintuitive negative direct association between trauma and personality pathology, which is not supported by existing theoretical or empirical literature.

Accordingly, we have revised the manuscript to ensure consistency across sections. We have removed the previous interpretation of suppression as a substantive mechanism and now emphasise that the association between adulthood betrayal trauma and PD severity appears to be carried almost entirely through indirect pathways (i.e., dissociation and ineffective mentalizing), which is consistent with the data. This more cautious way of presenting the results does not involve the assumption of a suppression effect, and we believe that this is sufficiently justified in the text.

Statistical Analyses :

Patterns consistent with statistical suppression (i.e., opposing signs of direct and indirect effects or indirect effects exceeding the total effect; MacKinnon et al., 2004) were noted descriptively and interpreted with caution, particularly in cases where direct effects were small or non-significant.

Results :

The single value exceeding 100% (observed in the pathway from adulthood betrayal trauma to PD severity) reflects a statistical artefact arising from a near-zero and oppositely signed direct effect (β = –.06, ns) combined with a substantial positive indirect effect (β = .27, $p < .001$). Such cases can produce proportions exceeding 100% and should not be interpreted as evidence of meaningful suppression, but rather as indicating that the total effect is almost entirely carried by indirect pathways.

(…)

Notably, the relationship between adulthood betrayal trauma and PD severity was characterised by a non-significant negative direct association (β = –.06) alongside positive indirect effects via mentalizing (β = 0.16) and dissociation (β = 0.11), resulting in a positive total effect (β = 0.21). This pattern indicates that the association operates almost entirely through these mediating mechanisms rather than directly. For adulthood betrayal trauma, indirect pathways accounted for 75.12% of the total effect on depressive symptoms and essentially all of the association with PD severity. Values exceeding 100% arise because the direct effect is very small and does not contribute meaningfully to the total effect.

The authors may consider tempering language around “full mediation” (pp. 29–30), given the cross-sectional design. Referring consistently to “statistical indirect effects” rather than mediation in a causal sense would maintain methodological rigor.

We thank the Reviewer for this important methodological clarification. We agree that the term “full mediation” may imply a stronger causal interpretation than warranted, particularly in a cross-sectional design. We have therefore revised the wording throughout the manuscript to avoid causal implications and to more precisely describe the observed pattern as indirect effects in the absence of significant direct effects, rather than “full mediation.” This phrasing more accurately reflects the statistical nature of our findings, as non-significant direct paths do not constitute evidence of their absence.

The authors may consider reporting confidence intervals for all total and direct effects in the main text tables, not only in supplementary materials, to enhance transparency.

We thank the reviewer for this helpful suggestion. To enhance transparency and facilitate interpretation of the results, we have revised the presentation of the main findings. Specifically, we moved the table containing total and direct effects from the Supplementary Materials to the main text and now report 95% confidence intervals for all estimates. In addition, we included unstandardized coefficients alongside standardized betas to support interpretability and future replication efforts.

The authors may consider further discussing the clinical implications of mentalizing emerging as the dominant pathway (pp. 33–36), particularly in relation to trauma-focused versus mentalization-based interventions. A clearer translation to treatment recommendations would enhance applied relevance.

Thank you for this comment, we have added the following sentences in the discussion section of the manuscript :

«More recently, a trauma-focused mentalization-based treatment approach has been developed to address the complex interplay of attachment disruptions, ineffective mentalizing and psychopathological outcomes related to what the authors address as attachment trauma (Smits et al., 2024), which can be considered as one of the forms that betrayal trauma can have (Bernstein & Freyd, 2014)."

[…]

« The therapeutic relationship can be considered a significant relationship which can foster the development of epistemic trust and reflective functioning, however a history of betrayals by significant figures can undermine the capacity to rely and trust even mental health professionals: A careful and precise assessment of trauma history can be considered as a fundamental part of the case formulation."

The authors may consider addressing alternative explanations, such as shared method variance due to exclusive reliance on self-report instruments, which may inflate associations between trauma, dissociation, mentalizing, and symptom measures.

Thank you for this comment, we have added the following sentence in the discussion section of the manuscript :

« Moreover, we can't exclude the possibility that shared method variance related to exclusive reliance on self-report instruments may inflate associations between trauma, dissociation, mentalizing, and symptom measures. »

The authors may consider integrating the suppression finding into broader developmental or resilience frameworks rather than framing it primarily as a statistical anomaly.

Please, see our answer above.

The authors may consider expanding the limitations section (pp. 37 onward) to more explicitly acknowledge:
The cross-sectional nature of the data and inability to establish temporal ordering.
Potential bidirectional relationships between depression and mentalizing deficits.
The possibility that current depressive state may bias retrospective trauma reporting.
The authors may consider proposing longitudinal or prospective designs to disentangle whether ineffective mentalizing precedes psychopathology or emerges as a consequence of it.

Authors' response: Thank you for this comment, we have modified the final section of the limitations.

It now is:

Finally, the cross-sectional design and use of mediation analyses preclude causal inference. Although the proposed mediation pathways are grounded in theory and supported by prior empirical evidence, these analyses cannot determine the temporal ordering of variables (Shrout & Bolger, 2002). Longitudinal and experimental designs are required to clarify causal mechanisms linking betrayal trauma, mentalizing, dissociation, and psychopathology.

The cross-sectional nature of the data does not allow us to exclude the possibility of bidirectional relationships between key variables. In particular, while ineffective mentalizing may contribute to the development or maintenance of depressive symptoms, it is also plausible that current depressive states impair reflective functioning, leading to elevated reports of mentalizing deficits. Longitudinal or prospective designs are therefore necessary to disentangle whether ineffective mentalizing precedes psychopathology or emerges as a consequence of it.

Additionally, the reliance on retrospective self-report measures of trauma introduces the possibility that current psychological states—particularly depressive symptoms—may influence the recall, interpretation, or reporting of past adverse experiences. Such mood-congruent recall biases may lead to either amplification or attenuation of reported trauma exposure, which should be taken into account when interpreting the observed associations.

The authors may consider discussing whether clinician-rated or performance-based mentalizing measures (e.g., RF interviews) might yield different patterns compared to self-report RFQ indices.

Thank you for this comment, this issue has been previously addressed in the literature on the assessment of mentalization, and we have added the following sentence in the manuscript :

« Moreover, we can't exclude the possibility that shared method variance related to exclusive reliance on self-report instruments may inflate associations between trauma, dissociation, mentalizing, and symptom measures. Jańczak (2021) and Hawksdale et al. (2025) have previously argued that different measures of mentalizing may address different relational contexts, with most measures assessing it in the context of a-specific relationships and only a smaller number of measures rating it in a more nuanced, and relational way. Mentalization is in fact a relational capacity that unfolds within the specific interpersonal context, and we can't exclude the possibility that different measures (e.g. clinician-report or interview-based measures) could lead to different patterns of relationships between variables. »

The authors may consider correcting minor typographical inconsistencies (e.g., spacing and punctuation in statistical reporting, occasional repetition in the Discussion).

We have carefully proofread the manuscript and corrected minor typographical inconsistencies.

The authors may consider ensuring consistent terminology throughout (e.g., "mentalizing deficits," "ineffective mentalizing," and "mentalizing difficulties" are used interchangeably).

Thank you for this comment, we have modified the manuscript and used 'ineffective mentalizing throughout all of it.
