# Supplementary material for: Betrayal trauma and adult mental health: The role of mentalizing and dissociation
Source: PLoS One. 2026 Jul 13;21(7):e0353662. doi: 10.1371/journal.pone.0353662 (PMC13362085; doi:10.1371/journal.pone.0353662)
Supplement: Attachment — Submitted filename: REV3_Response to Reviewers PLOS.pdf [file pone.0353662.s008.pdf]

PONE-D-25-62663
Betrayal Trauma and Adult Mental Health: The Mediating Role of Mentalizing and Dissociation
PLOS One

Dear Academic Editor and Reviewers,

We would like to sincerely thank the Academic Editor and both Reviewers for the time, effort, and thoughtful feedback they devoted to evaluating our manuscript. We greatly appreciate the constructive comments provided throughout the review process. We believe that the revised version is substantially stronger as a result. We are particularly grateful for the reviewers' positive evaluation of the revised manuscript and for acknowledging that the major concerns raised in previous review rounds have been satisfactorily addressed.
Following the remaining minor suggestions, we have carefully reviewed the manuscript once again and implemented additional refinements. Below, we provide a detailed point-by-point response to the remaining comments and describe all corresponding changes made in the manuscript.

Monika O. Jańczak, on behalf of all co-authors

Reviewer's comments

Rev#2
The manuscript is generally well written and theoretically informed, although some sections could still benefit from minor streamlining for readability. In several places, particularly within the introduction and discussion, the conceptual framing becomes somewhat dense due to the integration of multiple overlapping theoretical perspectives. While the material is relevant and demonstrates strong familiarity with the literature, a slightly more concise presentation would help maintain focus on the primary research questions and improve narrative flow. This is especially noticeable in sections discussing developmental cascades, epistemic trust, and neurodevelopmental mechanisms.

We thank the Reviewer for this helpful suggestion. In response, we carefully reviewed the Introduction and Discussion and made a number of minor revisions aimed at improving readability and narrative flow. Specifically, we streamlined several passages by reducing redundancy, simplifying theoretically dense formulations, and improving transitions between concepts. We also condensed several explanatory statements in the Discussion. These changes were intended solely to improve readability and narrative flow and do not affect the meaning, interpretation, or conclusions of the manuscript.We believe these revisions improve the clarity and accessibility of the manuscript while maintaining its conceptual depth.

The discussion of mentalizing has improved considerably following revision, particularly with the clarification that the RFQ_U primarily captures hypomentalizing rather than the full multidimensional construct of mentalizing. However, there remain a few instances where the manuscript refers broadly to "ineffective mentalizing" in ways that could be interpreted more expansively than the selected measure permits. A small amount of additional wording refinement may help ensure full alignment between theoretical claims and measurement specificity.

We particularly appreciate this comment, as conceptual clarity regarding mentalizing and its dimensions is a key focus of our broader research program. In response, we carefully reviewed the manuscript and

replaced the term “ineffective mentalizing” with the more precise term “hypomentalizing” (or uncertainty about mental states, where appropriate) in sections directly referring to the present study, including the aims, results, and discussion. We retained broader terminology only in parts of the Introduction that discuss previous research in which the specific dimension of mentalizing under investigation was not clearly specified. In addition, we included a brief definition of hypomentalizing in the Introduction to clarify the specific aspect of mentalizing assessed in the present study.
