# Supplementary material for: Betrayal trauma and adult mental health: The role of mentalizing and dissociation
Source: PLoS One. 2026 Jul 13;21(7):e0353662. doi: 10.1371/journal.pone.0353662 (PMC13362085; doi:10.1371/journal.pone.0353662)
Supplement: Peer review history

# Editor report: Decision Letter 0

**DOI:** 10.1371/journal.pone.0353662.r001

**Authors:** Hong Wang Fung

17 Dec 2025

PONE-D-25-62663

Betrayal Trauma and Adult Mental Health: The Mediating Role of Mentalizing and Dissociation

PLOS One

Dear Dr. Jańczak,

Thank you for submitting your manuscript to PLOS ONE. After careful consideration, we feel that it has merit but does not fully meet PLOS ONE’s publication criteria as it currently stands. Therefore, we invite you to submit a revised version of the manuscript that addresses the points raised during the review process.

Please submit your revised manuscript by Jan 31 2026 11:59PM. If you will need more time than this to complete your revisions, please reply to this message or contact the journal office at plosone@plos.org. When you're ready to submit your revision, log on to https://www.editorialmanager.com/pone/ and select the 'Submissions Needing Revision' folder to locate your manuscript file.

Please include the following items when submitting your revised manuscript:

- A letter that responds to each point raised by the academic editor and reviewer(s). You should upload this letter as a separate file labeled 'Response to Reviewers'.
- A marked-up copy of your manuscript that highlights changes made to the original version. You should upload this as a separate file labeled 'Revised Manuscript with Track Changes'.
- An unmarked version of your revised paper without tracked changes. You should upload this as a separate file labeled 'Manuscript'.

If you would like to make changes to your financial disclosure, please include your updated statement in your cover letter. Guidelines for resubmitting your figure files are available below the reviewer comments at the end of this letter.

If applicable, we recommend that you deposit your laboratory protocols in protocols.io to enhance the reproducibility of your results. Protocols.io assigns your protocol its own identifier (DOI) so that it can be cited independently in the future. For instructions see: https://journals.plos.org/plosone/s/submission-guidelines#loc-laboratory-protocols. Additionally, PLOS ONE offers an option for publishing peer-reviewed Lab Protocol articles, which describe protocols hosted on protocols.io. Read more information on sharing protocols at https://plos.org/protocols?utm_medium=editorial-email&utm_source=authorletters&utm_campaign=protocols.

We look forward to receiving your revised manuscript.

Kind regards,

Hong Wang Fung

Academic Editor

PLOS One

Journal Requirements:

When submitting your revision, we need you to address these additional requirements.

1. Please ensure that your manuscript meets PLOS ONE's style requirements, including those for file naming. The PLOS ONE style templates can be found at

https://journals.plos.org/plosone/s/file?id=wjVg/PLOSOne_formatting_sample_main_body.pdf and

https://journals.plos.org/plosone/s/file?id=ba62/PLOSOne_formatting_sample_title_authors_affiliations.pdf

2. Please include a complete copy of PLOS’ questionnaire on inclusivity in global research in your revised manuscript. Our policy for research in this area aims to improve transparency in the reporting of research performed outside of researchers’ own country or community. The policy applies to researchers who have travelled to a different country to conduct research, research with Indigenous populations or their lands, and research on cultural artefacts. The questionnaire can also be requested at the journal’s discretion for any other submissions, even if these conditions are not met.  Please find more information on the policy and a link to download a blank copy of the questionnaire here: https://journals.plos.org/plosone/s/best-practices-in-research-reporting. Please upload a completed version of your questionnaire as Supporting Information when you resubmit your manuscript.

3. Thank you for stating the following in the Acknowledgments Section of your manuscript:

“This research was funded by the Adam Mickiewicz University Program ID-UB “Initiative of Excellence – AMU Research University,” project no. 037/02/POB5/0027, awarded to MOJ (Monika Olga Jańczak). Funder website: https://amu.edu.pl Manuscript Click here to access/download;Manuscript;02_BODY_TraumaRelated Psychopathology.docx The funder had no role in study design, data collection and analysis, decision to publish, or preparation of the manuscript.”

We note that you have provided funding information that is not currently declared in your Funding Statement. However, funding information should not appear in the Acknowledgments section or other areas of your manuscript. We will only publish funding information present in the Funding Statement section of the online submission form.

Please remove any funding-related text from the manuscript and let us know how you would like to update your Funding Statement. Currently, your Funding Statement reads as follows:

“This research was funded by the Adam Mickiewicz University Program ID-UB “Initiative of Excellence – AMU Research University,” project no. 037/02/POB5/0027, awarded to MOJ (Monika Olga Jańczak). Funder website: https://amu.edu.pl The funder had no role in study design, data collection and analysis, decision to publish, or preparation of the manuscript.”

Please include your amended statements within your cover letter; we will change the online submission form on your behalf.

4. We note that the grant information you provided in the ‘Funding Information’ and ‘Financial Disclosure’ sections do not match.

When you resubmit, please ensure that you provide the correct grant numbers for the awards you received for your study in the ‘Funding Information’ section.

5. Thank you for stating the following financial disclosure:

“This research was funded by the Adam Mickiewicz University Program ID-UB “Initiative of Excellence – AMU Research University,” project no. 037/02/POB5/0027, awarded to MOJ (Monika Olga Jańczak). Funder website: https://amu.edu.pl The funder had no role in study design, data collection and analysis, decision to publish, or preparation of the manuscript.”

We note that one or more of the authors is affiliated with the funding organization, indicating the funder may have had some role in the design, data collection, analysis or preparation of your manuscript for publication; in other words, the funder played an indirect role through the participation of the co-authors. If the funding organization did not play a role in the study design, data collection and analysis, decision to publish, or preparation of the manuscript and only provided financial support in the form of authors' salaries and/or research materials, please do the following:

1. Review your statements relating to the author contributions, and ensure you have specifically and accurately indicated the role(s) that these authors had in your study. These amendments should be made in the online form.

2. Confirm in your cover letter that you agree with the following statement, and we will change the online submission form on your behalf:

“The funder provided support in the form of salaries for authors [insert relevant initials], but did not have any additional role in the study design, data collection and analysis, decision to publish, or preparation of the manuscript. The specific roles of these authors are articulated in the ‘author contributions’ section.

6. Please include captions for your Supporting Information files at the end of your manuscript, and update any in-text citations to match accordingly. Please see our Supporting Information guidelines for more information: http://journals.plos.org/plosone/s/supporting-information.

7. If the reviewer comments include a recommendation to cite specific previously published works, please review and evaluate these publications to determine whether they are relevant and should be cited. There is no requirement to cite these works unless the editor has indicated otherwise.

Additional Editor Comments:

Betrayal Trauma and Adult Mental Health: The Mediating Role of Mentalizing and Dissociation

PONE-D-25-62663

The topic of this study is interesting. It is a timely and important investigation. However, I do have several concerns and therefore would like to invite you to revise and resubmit before I send it out for peer review.

First of all, the relationship between betrayal trauma and dissociation / dissociative symptoms/features has been increasingly studied in the recent literature, but the authors failed to review the recent and most relevant literature. I would expect a more detailed, updated literature review, especially from the cross-cultural perspective, given the sample of your study.

Second, it is not acceptable and scientifically convincing enough to use cross-sectional data to explore or reveal mediating effects. Please reconsider what your data can and cannot tell.

[Note: HTML markup is below. Please do not edit.]

[NOTE: If reviewer comments were submitted as an attachment file, they will be attached to this email and accessible via the submission site. Please log into your account, locate the manuscript record, and check for the action link "View Attachments". If this link does not appear, there are no attachment files.]

To ensure your figures meet our technical requirements, please review our figure guidelines: https://journals.plos.org/plosone/s/figures

You may also use PLOS’s free figure tool, NAAS, to help you prepare publication quality figures: https://journals.plos.org/plosone/s/figures#loc-tools-for-figure-preparation.

NAAS will assess whether your figures meet our technical requirements by comparing each figure against our figure specifications.

# Author comment: Author response to Decision Letter 1

**DOI:** 10.1371/journal.pone.0353662.r002

5 Jan 2026

PONE-D-25-62663

Betrayal Trauma and Adult Mental Health: The Mediating Role of Mentalizing and Dissociation

PLOS One

We would like to sincerely thank the Editor for the careful evaluation of our manuscript and for recognizing the importance and timeliness of the topic. We appreciate the constructive feedback and the opportunity to revise and resubmit our work. Below, we provide a detailed, point-by-point response to all concerns raised and describe the revisions made to the manuscript accordingly.

Monika O. Jańczak, on behalf of all co-authors

Journal Requirements:

1. Please ensure that your manuscript meets PLOS ONE's style requirements, including those for file naming. The PLOS ONE style templates can be found at ..

We have revised the manuscript to ensure full compliance with PLOS ONE style and formatting requirements, including file naming conventions. All figures have been prepared and submitted in accordance with the journal’s technical specifications.

2. Please include a complete copy of PLOS’ questionnaire on inclusivity in global research in your revised manuscript. Our policy for research in this area aims to improve transparency in the reporting of research performed outside of researchers’ own country or community. The policy applies to researchers who have travelled to a different country to conduct research, research with Indigenous populations or their lands, and research on cultural artefacts. The questionnaire can also be requested at the journal’s discretion for any other submissions, even if these conditions are not met. Please find more information on the policy and a link to download a blank copy of the questionnaire here: https://journals.plos.org/plosone/s/best-practices-in-research-reporting. Please upload a completed version of your questionnaire as Supporting Information when you resubmit your manuscript.

We have included a completed copy of PLOS’ questionnaire on inclusivity in global research as Supporting Information with the revised manuscript, in accordance with the journal’s policy.

3. Thank you for stating the following in the Acknowledgments Section of your manuscript:

“This research was funded by the Adam Mickiewicz University Program ID-UB “Initiative of Excellence – AMU Research University,” project no. 037/02/POB5/0027, awarded to MOJ (Monika Olga Jańczak). Funder website: https://amu.edu.pl Manuscript Click here to access/download;Manuscript;02_BODY_TraumaRelated Psychopathology.docx The funder had no role in study design, data collection and analysis, decision to publish, or preparation of the manuscript.”

We note that you have provided funding information that is not currently declared in your Funding Statement. However, funding information should not appear in the Acknowledgments section or other areas of your manuscript. We will only publish funding information present in the Funding Statement section of the online submission form.

Please remove any funding-related text from the manuscript and let us know how you would like to update your Funding Statement. Currently, your Funding Statement reads as follows:

“This research was funded by the Adam Mickiewicz University Program ID-UB “Initiative of Excellence – AMU Research University,” project no. 037/02/POB5/0027, awarded to MOJ (Monika Olga Jańczak). Funder website: https://amu.edu.pl The funder had no role in study design, data collection and analysis, decision to publish, or preparation of the manuscript.”

Please include your amended statements within your cover letter; we will change the online submission form on your behalf.

Thank you for the clarification. We have removed all funding-related information from the manuscript.

We confirm that the Funding Statement should be as follows ::

“This research was funded by the Adam Mickiewicz University Program ID-UB ‘Initiative of Excellence – AMU Research University,’ project no. 037/02/POB5/0027, awarded to MOJ (Monika Olga Jańczak). Funder website: https://uczelniabadawcza.amu.edu.pl. The funder provided support in the form of salaries for author [MOJ], but did not have any additional role in the study design, data collection and analysis, decision to publish, or preparation of the manuscript. The specific roles of these authors are articulated in the ‘author contributions’ section.”

4. We note that the grant information you provided in the ‘Funding Information’ and ‘Financial Disclosure’ sections do not match.

When you resubmit, please ensure that you provide the correct grant numbers for the awards you received for your study in the ‘Funding Information’ section.

Thank you for noting this. We have reviewed and corrected the grant information to ensure that the ‘Funding Information’ section matches the ‘Financial Disclosure’ section.

5. Thank you for stating the following financial disclosure:

“This research was funded by the Adam Mickiewicz University Program ID-UB “Initiative of Excellence – AMU Research University,” project no. 037/02/POB5/0027, awarded to MOJ (Monika Olga Jańczak). Funder website: https://amu.edu.pl The funder had no role in study design, data collection and analysis, decision to publish, or preparation of the manuscript.”

We note that one or more of the authors is affiliated with the funding organization, indicating the funder may have had some role in the design, data collection, analysis or preparation of your manuscript for publication; in other words, the funder played an indirect role through the participation of the co-authors. If the funding organization did not play a role in the study design, data collection and analysis, decision to publish, or preparation of the manuscript and only provided financial support in the form of authors' salaries and/or research materials, please do the following:

1. Review your statements relating to the author contributions, and ensure you have specifically and accurately indicated the role(s) that these authors had in your study. These amendments should be made in the online form.

2. Confirm in your cover letter that you agree with the following statement, and we will change the online submission form on your behalf:

“The funder provided support in the form of salaries for authors [insert relevant initials], but did not have any additional role in the study design, data collection and analysis, decision to publish, or preparation of the manuscript. The specific roles of these authors are articulated in the ‘author contributions’ section.

With regard to the funding disclosure, we would like to clarify that the grant provided by the Adam Mickiewicz University Program ID-UB covered research-related costs only (e.g., participant compensation and recruitment expenses). No authors received salaries or personal remuneration from this funding source in relation to this project. The authors’ affiliation with the funding institution reflects standard academic employment and is not related to the funding of this project. The funder had no role in the study design, data collection and analysis, decision to publish, or preparation of the manuscript. We therefore confirm the Funding Statement as follows:

“This research was funded by the Adam Mickiewicz University Program ID-UB ‘Initiative of Excellence – AMU Research University,’ project no. 037/02/POB5/0027, awarded to MOJ (Monika Olga Jańczak). Funder website: https://uczelniabadawcza.amu.edu.pl. The funder provided support in the form of salaries for author [MOJ], but did not have any additional role in the study design, data collection and analysis, decision to publish, or preparation of the manuscript. The specific roles of these authors are articulated in the ‘author contributions’ section.”

6. Please include captions for your Supporting Information files at the end of your manuscript, and update any in-text citations to match accordingly. Please see our Supporting Information guidelines for more information: http://journals.plos.org/plosone/s/supporting-information.

We have added captions for all Supporting Information files at the end of the manuscript and updated the in-text citations accordingly to ensure consistency with PLOS ONE guidelines.

Additional Editor Comments:

The topic of this study is interesting. It is a timely and important investigation. However, I do have several concerns and therefore would like to invite you to revise and resubmit before I send it out for peer review.

We sincerely appreciate the Editor’s positive evaluation of the relevance and timeliness of our study. We are grateful for the opportunity to revise and resubmit the manuscript and have carefully addressed all concerns raised.

First of all, the relationship between betrayal trauma and dissociation / dissociative symptoms/features has been increasingly studied in the recent literature, but the authors failed to review the recent and most relevant literature. I would expect a more detailed, updated literature review, especially from the cross-cultural perspective, given the sample of your study.

We thank the Editor for this important comment and fully agree that the initial version of the manuscript did not sufficiently reflect the most recent and relevant literature on betrayal trauma and dissociation.

We acknowledge this as a significant oversight. In response, we have substantially revised the Introduction by adding a new paragraph that integrates recent empirical findings on the association between betrayal trauma and dissociation, including studies conducted in both English-speaking countries and culturally diverse contexts.

In addition, the cultural relevance of the current study has been more clearly articulated throughout the manuscript. Specifically, we have strengthened the cross-cultural framing in the Abstract, clarified this focus in the Aims of the study, and further elaborated on the contribution of data from an underrepresented Eastern European context in the Discussion.

Second, it is not acceptable and scientifically convincing enough to use cross-sectional data to explore or reveal mediating effects. Please reconsider what your data can and cannot tell.

Thank you for this important methodological comment. We fully agree that cross-sectional data do not allow for causal inference or for establishing temporal mediation mechanisms. In response, we have revised the manuscript to more clearly reflect what our data can support. Specifically, we have changed the title and removed the term “mediating” from all section headings, figure titles, and table titles, in order to avoid any implication that the identified pathways represent causal or developmental mechanisms. We have retained the use of mediation terminology in the statistical analysis section only, where it is explicitly framed as statistical mediation within a path-analytic framework, not as evidence of causal or temporal processes. Furthermore, references to mediation in the Aims, Abstract, and Discussion have been either removed or carefully reformulated to emphasize indirect associations and model-based pathways, with explicit clarification that these analyses are cross-sectional and exploratory in nature. We believe that these revisions appropriately align the interpretation of our findings with the limitations of the study design, while preserving a transparent and accurate description of the statistical methods employed.

Attachment Submitted filename: 02_PONE_Response Letter.pdf

# Peer review: Decision Letter 1

**DOI:** 10.1371/journal.pone.0353662.r003

**Authors:** Hong Wang Fung, Hong Wang Fung

9 Feb 2026

PONE-D-25-62663R1

Betrayal Trauma and Adult Mental Health: The Role of Mentalizing and Dissociation

PLOS One

Dear Dr. Jańczak,

Thank you for submitting your manuscript to PLOS ONE. After careful consideration, we feel that it has merit but does not fully meet PLOS ONE’s publication criteria as it currently stands. Therefore, we invite you to submit a revised version of the manuscript that addresses the points raised during the review process.

==============================

Our reviewers have recommended reconsideration after major revisions.

==============================

Please submit your revised manuscript by Mar 26 2026 11:59PM. If you will need more time than this to complete your revisions, please reply to this message or contact the journal office at plosone@plos.org. When you're ready to submit your revision, log on to https://www.editorialmanager.com/pone/ and select the 'Submissions Needing Revision' folder to locate your manuscript file.

Please include the following items when submitting your revised manuscript:

- A letter that responds to each point raised by the academic editor and reviewer(s). You should upload this letter as a separate file labeled 'Response to Reviewers'.
- A marked-up copy of your manuscript that highlights changes made to the original version. You should upload this as a separate file labeled 'Revised Manuscript with Track Changes'.
- An unmarked version of your revised paper without tracked changes. You should upload this as a separate file labeled 'Manuscript'.

If you would like to make changes to your financial disclosure, please include your updated statement in your cover letter. Guidelines for resubmitting your figure files are available below the reviewer comments at the end of this letter.

If applicable, we recommend that you deposit your laboratory protocols in protocols.io to enhance the reproducibility of your results. Protocols.io assigns your protocol its own identifier (DOI) so that it can be cited independently in the future. For instructions see: https://journals.plos.org/plosone/s/submission-guidelines#loc-laboratory-protocols. Additionally, PLOS ONE offers an option for publishing peer-reviewed Lab Protocol articles, which describe protocols hosted on protocols.io. Read more information on sharing protocols at https://plos.org/protocols?utm_medium=editorial-email&utm_source=authorletters&utm_campaign=protocols.

We look forward to receiving your revised manuscript.

Kind regards,

Hong Wang Fung

Academic Editor

PLOS One

Journal Requirements:

If the reviewer comments include a recommendation to cite specific previously published works, please review and evaluate these publications to determine whether they are relevant and should be cited. There is no requirement to cite these works unless the editor has indicated otherwise.

Additional Editor Comments:

Our reviewers have recommended reconsideration after major revisions.

[Note: HTML markup is below. Please do not edit.]

Reviewers' comments:

Reviewer's Responses to Questions

**Comments to the Author**

1. If the authors have adequately addressed your comments raised in a previous round of review and you feel that this manuscript is now acceptable for publication, you may indicate that here to bypass the “Comments to the Author” section, enter your conflict of interest statement in the “Confidential to Editor” section, and submit your "Accept" recommendation.

Reviewer #1: All comments have been addressed

**********

2. Is the manuscript technically sound, and do the data support the conclusions?

The manuscript must describe a technically sound piece of scientific research with data that supports the conclusions. Experiments must have been conducted rigorously, with appropriate controls, replication, and sample sizes. The conclusions must be drawn appropriately based on the data presented.

Reviewer #1: Yes

**********

3. Has the statistical analysis been performed appropriately and rigorously?

Reviewer #1: Yes

**********

4. Have the authors made all data underlying the findings in their manuscript fully available?

The PLOS Data policy requires authors to make all data underlying the findings described in their manuscript fully available without restriction, with rare exception (please refer to the Data Availability Statement in the manuscript PDF file). The data should be provided as part of the manuscript or its supporting information, or deposited to a public repository. For example, in addition to summary statistics, the data points behind means, medians and variance measures should be available. If there are restrictions on publicly sharing data—e.g. participant privacy or use of data from a third party—those must be specified.

Reviewer #1: Yes

**********

5. Is the manuscript presented in an intelligible fashion and written in standard English?

PLOS ONE does not copyedit accepted manuscripts, so the language in submitted articles must be clear, correct, and unambiguous. Any typographical or grammatical errors should be corrected at revision, so please note any specific errors here.

Reviewer #1: Yes

**********

6. Review Comments to the Author

Please use the space provided to explain your answers to the questions above. You may also include additional comments for the author, including concerns about dual publication, research ethics, or publication ethics. (Please upload your review as an attachment if it exceeds 20,000 characters)

Reviewer #1: Abstract

1. There is a slight inconsistency between the background and the objective. The background discusses the psychological mechanisms of trauma, while the objective focuses on the relationship between dissociation and ineffective mental mapping. It is recommended to revise this section to ensure consistency with the findings.

2. Regarding the participants, please briefly describe the source and duration of recruitment.

3. Because of the path analysis, please include each hypothetical path in the objective section.

Introduction

1. Since the researchers did not intend to measure resilience, it is recommended to change "Mentalizing and dissociation as resilience and risk factors following trauma" to "Mentalizing and dissociation as protective and risk factors following trauma."

2. The logic of the validation framework is unclear. It fails to infer why childhood betrayal trauma affects symptoms of personality disorders and depression, while adult betrayal trauma affects depressive symptoms. Which type of personality disorder does PD refer to? These should be explained in detail.

3. If a comparison of cultural differences is to be made, this study did not include participants from other cultures for comparison, so it is impossible to understand why cultural differences exist.

Overall, this topic is interesting. However, I could not fully understand about the rationale of the hypothesized paths in the present study.

**********

7. PLOS authors have the option to publish the peer review history of their article (what does this mean?). If published, this will include your full peer review and any attached files.

If you choose “no”, your identity will remain anonymous but your review may still be made public.

**Do you want your identity to be public for this peer review?**  For information about this choice, including consent withdrawal, please see our Privacy Policy.

Reviewer #1: **Yes:** Kunhua Lee

**********

[NOTE: If reviewer comments were submitted as an attachment file, they will be attached to this email and accessible via the submission site. Please log into your account, locate the manuscript record, and check for the action link "View Attachments". If this link does not appear, there are no attachment files.]

To ensure your figures meet our technical requirements, please review our figure guidelines: https://journals.plos.org/plosone/s/figures

You may also use PLOS’s free figure tool, NAAS, to help you prepare publication quality figures: https://journals.plos.org/plosone/s/figures#loc-tools-for-figure-preparation.

NAAS will assess whether your figures meet our technical requirements by comparing each figure against our figure specifications.

# Author comment: Author response to Decision Letter 2

**DOI:** 10.1371/journal.pone.0353662.r004

11 Feb 2026

PONE-D-25-62663

Betrayal Trauma and Adult Mental Health: The Mediating Role of Mentalizing and Dissociation

PLOS One

We sincerely thank the Reviewer for the careful and thorough evaluation of our manuscript and for acknowledging the relevance and timeliness of the topic. We are grateful for the constructive comments and the opportunity to revise and resubmit our work. Below, we provide a detailed, point-by-point response to all issues raised and outline the corresponding revisions made to the manuscript.

Monika O. Jańczak, on behalf of all co-authors

Reviewer’s comments:

#1: Abstract

1. There is a slight inconsistency between the background and the objective. The background discusses the psychological mechanisms of trauma, while the objective focuses on the relationship between dissociation and ineffective mental mapping. It is recommended to revise this section to ensure consistency with the findings.

We have revised the Abstract to make the link between the background and the study aims clearer and to ensure consistency with the Reviewer’s further comments regarding cultural differences.

2. Regarding the participants, please briefly describe the source and duration of recruitment.

The source and duration of participant recruitment have now been briefly described in the Abstract.

3. Because of the path analysis, please include each hypothetical path in the objective section.

Following the Reviewer’s suggestion, we have revised the Objective section to explicitly include the hypothesized paths tested in the path analysis.

Introduction

1. Since the researchers did not intend to measure resilience, it is recommended to change "Mentalizing and dissociation as resilience and risk factors following trauma" to "Mentalizing and dissociation as protective and risk factors following trauma."

We have revised the terminology throughout the manuscript to avoid implying that resilience was directly assessed. Following the Reviewer’s suggestion, we consistently use the terms “protective factors” and “risk factors” instead.

2. The logic of the validation framework is unclear. It fails to infer why childhood betrayal trauma affects symptoms of personality disorders and depression, while adult betrayal trauma affects depressive symptoms. Which type of personality disorder does PD refer to? These should be explained in detail.

We thank the Reviewer for highlighting the need to clarify the logic of the validation framework. In the revised manuscript, we have clarified this logic by more explicitly highlighting the arguments already presented in the Introduction. In particular, drawing on a developmental perspective on the timing of relational trauma, we emphasize that betrayal trauma occurring earlier in life is theorized to interfere with the formation of core self–other representations, attachment patterns, and regulatory capacities, thereby exerting more pervasive effects on personality functioning as well as mood-related symptoms. In contrast, betrayal trauma occurring in adulthood is assumed to impact an already consolidated personality structure, leading primarily to affective and symptomatic manifestations, such as depressive symptoms. We also adjusted the study aims accordingly.

We also clarify thorough the text that PD refers to the level of personality functioning within a dimensional PD model (as in ICD-11 and Alternative Model of PD in DSM-5), rather than to specific type of personality disorder. We also added a relevant line in the introduction.

3. If a comparison of cultural differences is to be made, this study did not include participants from other cultures for comparison, so it is impossible to understand why cultural differences exist.

We agree with the Reviewer that the present study does not allow for direct comparisons of cultural differences, as it does not include participants from multiple cultural contexts. In the revised manuscript, we have clarified this point and revised the text to avoid implying cross-cultural comparisons. We now frame the study as contributing data from an underrepresented population rather than making claims about cultural differences.

Overall, this topic is interesting. However, I could not fully understand about the rationale of the hypothesized paths in the present study.

We thank the Reviewer for this comment. We hope that the revisions made to the manuscript have clarified the rationale underlying the hypothesized paths.

Attachment Submitted filename: PONE_Response to Reviewers.pdf

# Peer review: Decision Letter 2

**DOI:** 10.1371/journal.pone.0353662.r005

**Authors:** Hong Wang Fung, Hong Wang Fung, Hong Wang Fung

28 Apr 2026

PONE-D-25-62663R2

Betrayal Trauma and Adult Mental Health: The Role of Mentalizing and Dissociation

PLOS One

Dear Dr. Jańczak,

Thank you for submitting your manuscript to PLOS ONE. After careful consideration, we feel that it has merit but does not fully meet PLOS ONE’s publication criteria as it currently stands. Therefore, we invite you to submit a revised version of the manuscript that addresses the points raised during the review process.

Please submit your revised manuscript by Jun 12 2026 11:59PM. If you will need more time than this to complete your revisions, please reply to this message or contact the journal office at plosone@plos.org. When you're ready to submit your revision, log on to https://www.editorialmanager.com/pone/ and select the 'Submissions Needing Revision' folder to locate your manuscript file.

Please include the following items when submitting your revised manuscript:

- A letter that responds to each point raised by the academic editor and reviewer(s). You should upload this letter as a separate file labeled 'Response to Reviewers'.
- A marked-up copy of your manuscript that highlights changes made to the original version. You should upload this as a separate file labeled 'Revised Manuscript with Track Changes'.
- An unmarked version of your revised paper without tracked changes. You should upload this as a separate file labeled 'Manuscript'.

If you would like to make changes to your financial disclosure, please include your updated statement in your cover letter. Guidelines for resubmitting your figure files are available below the reviewer comments at the end of this letter.

If applicable, we recommend that you deposit your laboratory protocols in protocols.io to enhance the reproducibility of your results. Protocols.io assigns your protocol its own identifier (DOI) so that it can be cited independently in the future. For instructions see: https://journals.plos.org/plosone/s/submission-guidelines#loc-laboratory-protocols. Additionally, PLOS ONE offers an option for publishing peer-reviewed Lab Protocol articles, which describe protocols hosted on protocols.io. Read more information on sharing protocols at https://plos.org/protocols?utm_medium=editorial-email&utm_source=authorletters&utm_campaign=protocols.

As the corresponding author, your ORCID iD is verified in the submission system and will appear in the published article. PLOS supports the use of ORCID, and we encourage all coauthors to register for an ORCID iD and use it as well. Please encourage your coauthors to verify their ORCID iD within the submission system before final acceptance, as unverified ORCID iDs will not appear in the published article. *Only*  the individual author can complete the verification step; PLOS staff *cannot*  verify ORCID iDs on behalf of authors.

We look forward to receiving your revised manuscript.

Kind regards,

Hong Wang Fung

Academic Editor

PLOS One

Journal Requirements:

If the reviewer comments include a recommendation to cite specific previously published works, please review and evaluate these publications to determine whether they are relevant and should be cited. There is no requirement to cite these works unless the editor has indicated otherwise.

Additional Editor Comments:

Our reviewers have recommended reconsideration after revisions. Please address their comments.

[Note: HTML markup is below. Please do not edit.]

Reviewers' comments:

Reviewer's Responses to Questions

**Comments to the Author**

1. If the authors have adequately addressed your comments raised in a previous round of review and you feel that this manuscript is now acceptable for publication, you may indicate that here to bypass the “Comments to the Author” section, enter your conflict of interest statement in the “Confidential to Editor” section, and submit your "Accept" recommendation.

Reviewer #1: All comments have been addressed

Reviewer #2: All comments have been addressed

**********

2. Is the manuscript technically sound, and do the data support the conclusions?

The manuscript must describe a technically sound piece of scientific research with data that supports the conclusions. Experiments must have been conducted rigorously, with appropriate controls, replication, and sample sizes. The conclusions must be drawn appropriately based on the data presented.

Reviewer #1: Partly

Reviewer #2: Yes

**********

3. Has the statistical analysis been performed appropriately and rigorously?

Reviewer #1: Yes

Reviewer #2: Yes

**********

4. Have the authors made all data underlying the findings in their manuscript fully available?

The PLOS Data policy requires authors to make all data underlying the findings described in their manuscript fully available without restriction, with rare exception (please refer to the Data Availability Statement in the manuscript PDF file). The data should be provided as part of the manuscript or its supporting information, or deposited to a public repository. For example, in addition to summary statistics, the data points behind means, medians and variance measures should be available. If there are restrictions on publicly sharing data—e.g. participant privacy or use of data from a third party—those must be specified.

Reviewer #1: Yes

Reviewer #2: Yes

**********

5. Is the manuscript presented in an intelligible fashion and written in standard English?

PLOS ONE does not copyedit accepted manuscripts, so the language in submitted articles must be clear, correct, and unambiguous. Any typographical or grammatical errors should be corrected at revision, so please note any specific errors here.

Reviewer #1: Yes

Reviewer #2: Yes

**********

6. Review Comments to the Author

Please use the space provided to explain your answers to the questions above. You may also include additional comments for the author, including concerns about dual publication, research ethics, or publication ethics. (Please upload your review as an attachment if it exceeds 20,000 characters)

Reviewer #1: Thanks for considering my comments and suggestions. The revised manuscript is more comprehensive. Only one suggestion is to consider the necessarity of "neurobiological mechanisms of trauma". Actually, the present study did not collect the biological data, such as neuroimage or biological markers. Please to focus on the psychological mechanism of trauma.

Reviewer #2: The authors may consider further strengthening the conceptual clarity of the manuscript by tightening the alignment between the theoretical framework (developmental psychopathology and betrayal trauma theory) and the specific statistical model tested. While the cascade model is well described, the justification for positioning dissociation and mentalizing strictly as parallel (rather than sequential or interacting) mediators could be elaborated further.

The authors may consider clarifying the conceptual distinction between “ineffective mentalizing” and “hypomentalizing,” particularly given that only the RFQ_U subscale was used (pp. 22–23). A brief rationale for excluding RFQ_C and a discussion of the implications for construct coverage would strengthen interpretability.

The authors may consider more explicitly articulating the theoretical rationale for including both childhood and adulthood betrayal trauma in the same model, especially given their moderate covariance (Table 1; pp. 27–28). Clarifying whether adulthood trauma is conceptualized as independent exposure, continuation, or potential revictimization would improve theoretical precision.

The authors may consider streamlining the neurobiological section (pp. 14–15) to focus more directly on mechanisms that are empirically linked to dissociation and mentalizing. While comprehensive, some neuroimaging findings appear somewhat distal to the specific mediation model tested.

The authors may consider more clearly differentiating between betrayal trauma and broader interpersonal trauma constructs early in the introduction. Although betrayal trauma theory is referenced, a sharper operational definition would help readers understand how it uniquely informs the hypotheses.

The authors may consider clarifying the logic underlying the hypothesis that adulthood trauma would primarily relate to depression but not PD severity (pp. 18–19), particularly given that the results showed comparable total effects on PD severity. A more cautious phrasing of the hypothesis or a clearer developmental argument may be warranted.

The authors may consider providing more detail regarding the Polish translation of the BBTS (p. 22). Specifically, information about translation procedures (e.g., back-translation, pilot testing) would strengthen confidence in measurement validity.

The authors may consider reporting the proportion and pattern of missing data prior to missForest imputation (p. 24). Transparency regarding the percentage of missingness per variable would enhance methodological clarity.

The authors may consider clarifying whether recruitment source (clinical vs. community; p. 21) was tested as a moderator or covariate. Given the mixed sample, subgroup differences could meaningfully influence associations.

The authors may consider justifying the exclusion of adolescence trauma indices from the BBTS (p. 22), especially as adolescence represents a developmentally sensitive period relevant to personality functioning.

Statistical Analysis and Interpretation

The authors may consider presenting unstandardized coefficients alongside standardized betas in supplementary materials to facilitate replication and meta-analytic integration.

The authors may consider elaborating on the suppression effect observed in the adulthood trauma–PD pathway (pp. 30–31). Although described as a statistical artefact, a more nuanced theoretical reflection on possible interpretative scenarios would add depth.

The authors may consider tempering language around “full mediation” (pp. 29–30), given the cross-sectional design. Referring consistently to “statistical indirect effects” rather than mediation in a causal sense would maintain methodological rigor.

The authors may consider reporting confidence intervals for all total and direct effects in the main text tables, not only in supplementary materials, to enhance transparency.

The authors may consider further discussing the clinical implications of mentalizing emerging as the dominant pathway (pp. 33–36), particularly in relation to trauma-focused versus mentalization-based interventions. A clearer translation to treatment recommendations would enhance applied relevance.

The authors may consider addressing alternative explanations, such as shared method variance due to exclusive reliance on self-report instruments, which may inflate associations between trauma, dissociation, mentalizing, and symptom measures.

The authors may consider integrating the suppression finding into broader developmental or resilience frameworks rather than framing it primarily as a statistical anomaly.

The authors may consider expanding the limitations section (pp. 37 onward) to more explicitly acknowledge:

The cross-sectional nature of the data and inability to establish temporal ordering.

Potential bidirectional relationships between depression and mentalizing deficits.

The possibility that current depressive state may bias retrospective trauma reporting.

The authors may consider proposing longitudinal or prospective designs to disentangle whether ineffective mentalizing precedes psychopathology or emerges as a consequence of it.

The authors may consider discussing whether clinician-rated or performance-based mentalizing measures (e.g., RF interviews) might yield different patterns compared to self-report RFQ indices.

The authors may consider correcting minor typographical inconsistencies (e.g., spacing and punctuation in statistical reporting, occasional repetition in the Discussion).

The authors may consider ensuring consistent terminology throughout (e.g., “mentalizing deficits,” “ineffective mentalizing,” and “mentalizing difficulties” are used interchangeably).

**********

7. PLOS authors have the option to publish the peer review history of their article (what does this mean?). If published, this will include your full peer review and any attached files.

If you choose “no”, your identity will remain anonymous but your review may still be made public.

**Do you want your identity to be public for this peer review?**  For information about this choice, including consent withdrawal, please see our Privacy Policy.

Reviewer #1: No

Reviewer #2: **Yes:** Peejay Bengwasan

**********

[NOTE: If reviewer comments were submitted as an attachment file, they will be attached to this email and accessible via the submission site. Please log into your account, locate the manuscript record, and check for the action link "View Attachments". If this link does not appear, there are no attachment files.]

To ensure your figures meet our technical requirements, please review our figure guidelines: https://journals.plos.org/plosone/s/figures

You may also use PLOS’s free figure tool, NAAS, to help you prepare publication quality figures: https://journals.plos.org/plosone/s/figures#loc-tools-for-figure-preparation.

NAAS will assess whether your figures meet our technical requirements by comparing each figure against our figure specifications.

# Author comment: Author response to Decision Letter 3

**DOI:** 10.1371/journal.pone.0353662.r006

5 May 2026

For improved readability, we kindly invite you to see the attached version of the responses.

PONE-D-25-62663

Betrayal Trauma and Adult Mental Health: The Mediating Role of Mentalizing and Dissociation

PLOS One

We sincerely thank the Reviewers for the careful and thorough evaluation of our manuscript and for acknowledging the relevance and timeliness of the topic. We are grateful for the constructive comments and the opportunity to revise and resubmit our work. Below, we provide a detailed, point-by-point response to all issues raised and outline the corresponding revisions made to the manuscript. All changes have been implemented in the revised version and are visible using tracked changes. Where feasible, we have included excerpts of the revisions directly in our responses; however, longer or more dispersed changes are indicated only in the tracked manuscript.

Monika O. Jańczak, on behalf of all co-authors

Reviewer’s comments

Reviewer #1: Thanks for considering my comments and suggestions. The revised manuscript is more comprehensive. Only one suggestion is to consider the necessarity of "neurobiological mechanisms of trauma". Actually, the present study did not collect the biological data, such as neuroimage or biological markers. Please to focus on the psychological mechanism of trauma.

Thank you for this helpful suggestion. In response to this comment and the comment of Reviewer 2, we have substantially shortened the section on neurobiological mechanisms and retained only a minimal reference to neurodevelopmental evidence to support the possible distinct consequences between childhood and adulthood trauma.

Reviewer #2: The authors may consider further strengthening the conceptual clarity of the manuscript by tightening the alignment between the theoretical framework (developmental psychopathology and betrayal trauma theory) and the specific statistical model tested. While the cascade model is well described, the justification for positioning dissociation and mentalizing strictly as parallel (rather than sequential or interacting) mediators could be elaborated further.

Thank you for this comment. In response, we have revised the Aims section to more explicitly align the statistical model with the theoretical framework discussed in the Introduction regarding the relationship between mentalizing and dissociation.

It is now at follows:

Aims:

It was further hypothesised that higher levels of betrayal trauma would correspond with greater ineffective mentalizing and higher dissociation, particularly for childhood trauma. Ineffective mentalizing and dissociation were expected to show positive associations with both depression and PD severity. Finally, consistent with the developmental psychopathology framework proposed by Bateman and Fonagy (2019), it was anticipated that ineffective mentalizing would be positively correlated with dissociation. Given the mixed and bidirectional findings in prior research, and the lack of consistent evidence supporting a unidirectional pathway, the present study modelled dissociation and mentalizing as parallel mediators, capturing their potentially independent yet interrelated contributions to trauma-related psychopathology.

The authors may consider clarifying the conceptual distinction between “ineffective mentalizing” and “hypomentalizing,” particularly given that only the RFQ_U subscale was used (pp. 22–23). A brief rationale for excluding RFQ_C and a discussion of the implications for construct coverage would strengthen interpretability.

We agree that ineffective mentalizing is a multidimensional construct that may manifest in different forms, including both hypomentalizing (uncertainty about mental states) and hypermentalizing (overinterpretation or unwarranted certainty). Moreover, recent research suggests that distinctions such as self–other imbalance and hyper- versus hypomentalizing may differentially shape clinical presentations and psychopathology. Therefore, it is important to explicitly specify which facet of mentalizing is captured in the present study.

In this study, we focused on the RFQ_U subscale as an index of hypomentalizing, reflecting uncertainty and a reduced capacity to infer mental states. This decision was guided both by theoretical and methodological considerations. From a theoretical perspective, impairments in self-focused mentalizing appear to play a particularly central role in psychopathology, especially in relation to affect regulation and vulnerability to depressive and personality pathology (Fonagy, Bateman, 2019; Jańczak et al., 2025). From a methodological standpoint, prior validation studies indicate that RFQ_U demonstrates more robust psychometric properties and clearer interpretability compared to RFQ_C, which has been shown to be more difficult to interpret and may capture heterogeneous processes, including both adaptive certainty and maladaptive overconfidence (Müller et al., 2020; Woźniak-Prus et al., 2022).

At the same time, we acknowledge that relying exclusively on RFQ_U limits the scope of mentalizing assessment, as it does not capture hypermentalizing or more nuanced self–other distinctions. This constraint may have reduced our ability to detect differential patterns associated with specific forms of mentalizing dysfunction. We have now clarified this issue in the Methods section and elaborated on its implications in the Discussion.

Methods

Reflective Functioning Questionnaire (RFQ-8; Fonagy et al., 2016). The RFQ-8 is an eight-item self-report measure of mentalizing. Each item is rated on a seven-point Likert scale (1 = strongly disagree to 7 = strongly agree). The questionnaire includes two subscales: certainty (RFQ_C) and uncertainty (RFQ_U) about mental states. In the present study, we focused on the RFQ_U subscale, which primarily captures hypomentalizing, defined as uncertainty about mental states, particularly in relation to the self. This decision was based on prior psychometric evidence indicating that RFQ_U demonstrates more robust properties and clearer interpretability compared to RFQ_C, which has been questioned as a valid indicator of ineffective mentalizing (e.g., Müller et al., 2020; Woźniak-Prus et al., 2022). In addition, focusing exclusively on RFQ_U avoids methodological issues related to double-scoring and the mathematical dependence between the two subscales, while remaining consistent with more recent unidimensional approaches to RFQ scoring (Müller et al., 2020; Woźniak-Prus et al., 2022). This approach has also been adopted in recent studies using the RFQ (e.g., Bateman et al., 2023). The validated Polish version (Woźniak-Prus et al., 2022) was used and demonstrated acceptable internal consistency in the present sample (Cronbach’s α = 0.79).

Discussion:

It is important to note that the mentalizing measure used in the present study primarily captures hypomentalizing, defined as uncertainty about mental states, particularly in relation to the self. However, ineffective mentalizing is a multidimensional construct that may take different forms. Recent research highlights that distinctions between self- and other-focused mentalizing, as well as between hypo- and hypermentalizing, may be especially important in shaping clinical presentations (Barnby et al., 2025; Jańczak et al., 2025; Jańczak et. al. 2026). Accordingly, the present study does not capture other clinically relevant forms of mentalizing dysfunction, such as hypermentalizing or imbalances between self- and other-focused processing. These distinct patterns may be associated with different symptom profiles and mechanisms of psychopathology, and future research should aim to disentangle their specific contributions using more comprehensive and multi-method assessments.

The authors may consider more explicitly articulating the theoretical rationale for including both childhood and adulthood betrayal trauma in the same model, especially given their moderate covariance (Table 1; pp. 27–28). Clarifying whether adulthood trauma is conceptualized as independent exposure, continuation, or potential revictimization would improve theoretical precision.

We agree that clarifying the conceptual rationale for including both childhood and adulthood betrayal trauma in the same model strengthens the theoretical precision of the study.

In the present framework, childhood and adulthood betrayal trauma were conceptualised as developmentally distinct but potentially related exposures. Childhood trauma was treated as an early vulnerability factor that may shape the development of mentalizing capacities and dissociative tendencies, thereby exerting long-term effects on personality pathology and emotional functioning. In contrast, adulthood trauma was conceptualised as a more proximal stressor, reflecting either independent exposure or, in some cases, potential revictimization linked to earlier vulnerabilities.

The inclusion of both indices in a single model was intended to disentangle their unique and shared contributions to psychopathology, while accounting for their natural co-occurrence. The observed covariance between childhood and adulthood trauma is consistent with prior research on revictimization and cumulative adversity and was therefore modelled explicitly rather than treated as a confound.

We have now clarified this rationale in the Introduction and Discussion sections of the manuscript.

The authors may consider streamlining the neurobiological section (pp. 14–15) to focus more directly on mechanisms that are empirically linked to dissociation and mentalizing. While comprehensive, some neuroimaging findings appear somewhat distal to the specific mediation model tested.

Thank you for this helpful suggestion. In response to this comment and the comment of Reviewer 1, we have substantially shortened the section on neurobiological mechanisms and retained only a minimal reference to neurodevelopmental evidence to support the possible distinct consequences between childhood and adulthood trauma.

The authors may consider more clearly differentiating between betrayal trauma and broader interpersonal trauma constructs early in the introduction. Although betrayal trauma theory is referenced, a sharper operational definition would help readers understand how it uniquely informs the hypotheses.

In line with this suggestion, we have revised the Introduction to provide a more explicit operational definition of betrayal trauma.

Introduction:

Complementing this, betrayal trauma theory differentiates adverse experiences by the degree of betrayal involved: maltreatment perpetrated by a trusted other within a relationship of attachment or dependency constitutes a specific subtype of interpersonal trauma, distinct from harm inflicted by non-significant others (Goldberg & Freyd, 2006; Freyd, 1994). Such experiences may entail unique adaptational demands.

The authors may consider clarifying the logic underlying the hypothesis that adulthood trauma would primarily relate to depression but not PD severity (pp. 18–19), particularly given that the results showed comparable total effects on PD severity. A more cautious phrasing of the hypothesis or a clearer developmental argument may be warranted.

We thank the Reviewer for this helpful comment. In response, we have expanded the rationale in the Aims section to further clarify the developmental logic underlying our hypothesis. We have also revised the Discussion to more explicitly acknowledge that this hypothesis was only partially supported, and to more clearly address the discrepancy between the expected and observed patterns, which was not sufficiently highlighted in the previous version of the manuscript.

Aims:

In contrast, given that personality structure is largely consolidated by adulthood, trauma occurring later in life may function more as a proximal stressor linked to depressive symptoms than as a factor shaping enduring personality pathology.

Discussion:

This hypothesis was only partially supported, as adulthood trauma showed comparable total effects on both depressive symptoms and PD severity. This finding suggests that trauma occurring in adulthood may not be limited to symptomatic outcomes but may also be associated with personality functioning. One possible explanation is that adulthood trauma may activate pre-existing vulnerabilities shaped by earlier developmental experiences or reflect cumulative exposure processes, rather than operating independently of personality structure.

The authors may consider providing more detail regarding the Polish translation of the BBTS (p. 22). Specifically, information about translation procedures (e.g., back-translation, pilot testing) would strengthen confidence in measurement validity.

We agree that providing additional detail on the translation procedure strengthens confidence in the validity of the measure. We have now expanded the description of the Polish adaptation of the BBTS in the Methods section. A more detailed description of the adaptation and validation procedure is provided in a separate manuscript currently in preparation (Sienski & Jańczak, in preparation), which we now cite in the text.

Methods:

A Polish translation of the BBTS was developed using a multi-step procedure. Three independent forward translations were prepared by experienced clinicians with advanced proficiency in English, followed by expert panel reconciliation. The reconciled version was then back-translated into English by an independent translator experienced in academic translation and compared with the original instrument to ensure semantic and conceptual equivalence. The final version was reviewed in consultation with the original author of the instrument (J. Freyd). A more detailed description of the adaptation and validation process is provided elsewhere (Sienski & Jańczak, in preparation).

The authors may consider reporting the proportion and pattern of missing data prior to missForest imputation (p. 24). Transparency regarding the percentage of missingness per variable would enhance methodological clarity.

We thank the reviewer for this helpful comment. We have now clarified the proportion and pattern of missing data in the Methods section. The overall proportion of missing data was low (1.10%), and no systematic pattern of missingness was observed.

The authors may consider clarifying whether recruitment source (clinical vs. community; p. 21) was tested as a moderator or covariate. Given the mixed sample, subgroup differences could meaningfully influence associations.

We appreciate this important comment. The present study was designed as a dimensional, transdiagnostic investigation rather than a categorical comparison of recruitment groups. Accordingly, participants were recruited from both clinical and community settings to maximise variability in trauma exposure, personality pathology, and depressive symptoms, thereby enabling the examination of mechanisms across a broader severity continuum. This dimensional approach is consistent with contemporary frameworks of psychopathology and constitutes a strength of the study, as it allows for testing associations across naturally occurring variability rather than within restricted subgroups.

At the same time, we acknowledge that recruitment source may represent a potential source of heterogeneity, and we agree that such differences should be taken into account to improve the interpretability of the findings. Therefore, in response to the reviewer’s comment and to strengthen the robustness of our conclusions, we conducted additional exploratory analyses to examine whether recruitment source alters the structural relations of interest.

To address potential heterogeneity related to recruitment source, we conducted an exploratory moderation analysis testing whether recruitment source (clinical vs. community sample) moderated the associations between betrayal trauma and the mediators. Interaction effects were not supported by bootstrap estimates. Specifically, the interaction between childhood trauma and recruitment source predicting dissociation was non-significant (B = 6.06, β = .09, p = .380, 95% CI [−7.714, 19.406]), as was the corresponding interaction for adulthood trauma (B = −0.26, β = −.00, p = .979, 95% CI [−21.127, 19.245]). Similarl

Attachment Submitted filename: REV2_Response to Reviewers PLOS.pdf

# Peer review: Decision Letter 3

**DOI:** 10.1371/journal.pone.0353662.r007

**Authors:** Hong Wang Fung, Hong Wang Fung, Hong Wang Fung, Hong Wang Fung

2 Jun 2026

PONE-D-25-62663R3

Betrayal Trauma and Adult Mental Health: The Role of Mentalizing and Dissociation

PLOS One

Dear Dr. Jańczak,

Thank you for submitting your manuscript to PLOS ONE. After careful consideration, we feel that it has merit but does not fully meet PLOS ONE’s publication criteria as it currently stands. Therefore, we invite you to submit a revised version of the manuscript that addresses the points raised during the review process.

**Please address the reviewers' comments.**

Please submit your revised manuscript by Jul 17 2026 11:59PM. If you will need more time than this to complete your revisions, please reply to this message or contact the journal office at plosone@plos.org. When you're ready to submit your revision, log on to https://www.editorialmanager.com/pone/ and select the 'Submissions Needing Revision' folder to locate your manuscript file.

Please include the following items when submitting your revised manuscript:

- A letter that responds to each point raised by the academic editor and reviewer(s). You should upload this letter as a separate file labeled 'Response to Reviewers'.
- A marked-up copy of your manuscript that highlights changes made to the original version. You should upload this as a separate file labeled 'Revised Manuscript with Track Changes'.
- An unmarked version of your revised paper without tracked changes. You should upload this as a separate file labeled 'Manuscript'.

If you would like to make changes to your financial disclosure, please include your updated statement in your cover letter. Guidelines for resubmitting your figure files are available below the reviewer comments at the end of this letter.

If applicable, we recommend that you deposit your laboratory protocols in protocols.io to enhance the reproducibility of your results. Protocols.io assigns your protocol its own identifier (DOI) so that it can be cited independently in the future. For instructions see: https://journals.plos.org/plosone/s/submission-guidelines#loc-laboratory-protocols. Additionally, PLOS ONE offers an option for publishing peer-reviewed Lab Protocol articles, which describe protocols hosted on protocols.io. Read more information on sharing protocols at https://plos.org/protocols?utm_medium=editorial-email&utm_source=authorletters&utm_campaign=protocols.

As the corresponding author, your ORCID iD is verified in the submission system and will appear in the published article. PLOS supports the use of ORCID, and we encourage all coauthors to register for an ORCID iD and use it as well. Please encourage your coauthors to verify their ORCID iD within the submission system before final acceptance, as unverified ORCID iDs will not appear in the published article. *Only*  the individual author can complete the verification step; PLOS staff *cannot*  verify ORCID iDs on behalf of authors.

We look forward to receiving your revised manuscript.

Kind regards,

Hong Wang Fung

Academic Editor

PLOS One

**Journal Requirements:**

1. If the reviewer comments include a recommendation to cite specific previously published works, please review and evaluate these publications to determine whether they are relevant and should be cited. There is no requirement to cite these works unless the editor has indicated otherwise.

2. Please review your reference list to ensure that it is complete and correct. If you have cited papers that have been retracted, please include the rationale for doing so in the manuscript text, or remove these references and replace them with relevant current references. Any changes to the reference list should be mentioned in the rebuttal letter that accompanies your revised manuscript. If you need to cite a retracted article, indicate the article’s retracted status in the References list and also include a citation and full reference for the retraction notice.

**Additional Editor Comments:**

Please address the reviewers' comments.

[Note: HTML markup is below. Please do not edit.]

Reviewers' comments:

Reviewer's Responses to Questions

**Comments to the Author**

1. If the authors have adequately addressed your comments raised in a previous round of review and you feel that this manuscript is now acceptable for publication, you may indicate that here to bypass the “Comments to the Author” section, enter your conflict of interest statement in the “Confidential to Editor” section, and submit your "Accept" recommendation.

Reviewer #1: All comments have been addressed

Reviewer #2: All comments have been addressed

**********

2. Is the manuscript technically sound, and do the data support the conclusions?

The manuscript must describe a technically sound piece of scientific research with data that supports the conclusions. Experiments must have been conducted rigorously, with appropriate controls, replication, and sample sizes. The conclusions must be drawn appropriately based on the data presented.

Reviewer #1: Yes

Reviewer #2: Yes

**********

3. Has the statistical analysis been performed appropriately and rigorously?

Reviewer #1: Yes

Reviewer #2: Yes

**********

4. Have the authors made all data underlying the findings in their manuscript fully available?

The PLOS Data policy requires authors to make all data underlying the findings described in their manuscript fully available without restriction, with rare exception (please refer to the Data Availability Statement in the manuscript PDF file). The data should be provided as part of the manuscript or its supporting information, or deposited to a public repository. For example, in addition to summary statistics, the data points behind means, medians and variance measures should be available. If there are restrictions on publicly sharing data—e.g. participant privacy or use of data from a third party—those must be specified.

Reviewer #1: Yes

Reviewer #2: Yes

**********

5. Is the manuscript presented in an intelligible fashion and written in standard English?

PLOS ONE does not copyedit accepted manuscripts, so the language in submitted articles must be clear, correct, and unambiguous. Any typographical or grammatical errors should be corrected at revision, so please note any specific errors here.

Reviewer #1: Yes

Reviewer #2: Yes

**********

6. Review Comments to the Author

Please use the space provided to explain your answers to the questions above. You may also include additional comments for the author, including concerns about dual publication, research ethics, or publication ethics. (Please upload your review as an attachment if it exceeds 20,000 characters)

**Reviewer #1:** Thanks for considering my suggestions, and I have no other comments. This manuscript is comprehensive to be published.

**Reviewer #2:**  The manuscript presents a thoughtful and clinically meaningful examination of betrayal trauma, dissociation, and ineffective mentalizing within a dimensional framework of psychopathology. The study addresses an important gap in the literature by integrating betrayal trauma theory with developmental psychopathology and contemporary ICD-11 approaches to personality functioning. The focus on psychological mechanisms rather than solely exposure-outcome associations strengthens the conceptual contribution of the work, particularly within trauma and personality research. The manuscript is additionally strengthened by its transparent reporting practices, openly available data, and inclusion of a relatively underrepresented Eastern European sample.

The revised version demonstrates substantial responsiveness to prior reviewer feedback. The authors have improved the conceptual clarity of the manuscript, particularly regarding the rationale for modelling dissociation and ineffective mentalizing as parallel mediators, the distinction between hypomentalizing and broader mentalizing difficulties, and the developmental reasoning underlying childhood versus adulthood trauma pathways. The additional methodological clarifications regarding recruitment source, missing data, and statistical interpretation also improve the overall rigor and transparency of the paper. The decision to temper causal language surrounding mediation effects is especially appropriate given the cross-sectional nature of the design.

The manuscript is generally well written and theoretically informed, although some sections could still benefit from minor streamlining for readability. In several places, particularly within the introduction and discussion, the conceptual framing becomes somewhat dense due to the integration of multiple overlapping theoretical perspectives. While the material is relevant and demonstrates strong familiarity with the literature, a slightly more concise presentation would help maintain focus on the primary research questions and improve narrative flow. This is especially noticeable in sections discussing developmental cascades, epistemic trust, and neurodevelopmental mechanisms.

The discussion of mentalizing has improved considerably following revision, particularly with the clarification that the RFQ_U primarily captures hypomentalizing rather than the full multidimensional construct of mentalizing. However, there remain a few instances where the manuscript refers broadly to “ineffective mentalizing” in ways that could be interpreted more expansively than the selected measure permits. A small amount of additional wording refinement may help ensure full alignment between theoretical claims and measurement specificity.

The authors appropriately acknowledge the limitations associated with the exclusive reliance on self-report instruments and the cross-sectional design. The revised limitations section now provides a balanced discussion of bidirectionality, recall bias, and temporal ambiguity, which strengthens the methodological integrity of the paper.

Overall, this is a strong and carefully revised manuscript that makes a valuable contribution to the literature on betrayal trauma and trauma-related psychopathology. The findings regarding ineffective mentalizing as a particularly salient indirect pathway are clinically relevant and may have meaningful implications for trauma-informed and mentalization-based interventions.

**********

7. PLOS authors have the option to publish the peer review history of their article (what does this mean?). If published, this will include your full peer review and any attached files.

If you choose “no”, your identity will remain anonymous but your review may still be made public.

**Do you want your identity to be public for this peer review?**  For information about this choice, including consent withdrawal, please see our Privacy Policy.

Reviewer #1: **Yes:** Kunhua Lee

Reviewer #2: **Yes:** Peejay D. Bengwasan

**********

[NOTE: If reviewer comments were submitted as an attachment file, they will be attached to this email and accessible via the submission site. Please log into your account, locate the manuscript record, and check for the action link "View Attachments". If this link does not appear, there are no attachment files.]

To ensure your figures meet our technical requirements, please review our figure guidelines: https://journals.plos.org/plosone/s/figures

You may also use PLOS’s free figure tool, NAAS, to help you prepare publication quality figures: https://journals.plos.org/plosone/s/figures#loc-tools-for-figure-preparation.

NAAS will assess whether your figures meet our technical requirements by comparing each figure against our figure specifications.

# Author comment: Author response to Decision Letter 4

**DOI:** 10.1371/journal.pone.0353662.r008

3 Jun 2026

PONE-D-25-62663

Betrayal Trauma and Adult Mental Health: The Mediating Role of Mentalizing and Dissociation

PLOS One

Dear Academic Editor and Reviewers,

We would like to sincerely thank the Academic Editor and both Reviewers for the time, effort, and thoughtful feedback they devoted to evaluating our manuscript. We greatly appreciate the constructive comments provided throughout the review process. We believe that the revised version is substantially stronger as a result. We are particularly grateful for the reviewers’ positive evaluation of the revised manuscript and for acknowledging that the major concerns raised in previous review rounds have been satisfactorily addressed.

Following the remaining minor suggestions, we have carefully reviewed the manuscript once again and implemented additional refinements. Below, we provide a detailed point-by-point response to the remaining comments and describe all corresponding changes made in the manuscript.

Monika O. Jańczak, on behalf of all co-authors

Reviewer’s comments

Rev#2

The manuscript is generally well written and theoretically informed, although some sections could still benefit from minor streamlining for readability. In several places, particularly within the introduction and discussion, the conceptual framing becomes somewhat dense due to the integration of multiple overlapping theoretical perspectives. While the material is relevant and demonstrates strong familiarity with the literature, a slightly more concise presentation would help maintain focus on the primary research questions and improve narrative flow. This is especially noticeable in sections discussing developmental cascades, epistemic trust, and neurodevelopmental mechanisms.

We thank the Reviewer for this helpful suggestion. In response, we carefully reviewed the Introduction and Discussion and made a number of minor revisions aimed at improving readability and narrative flow. Specifically, we streamlined several passages by reducing redundancy, simplifying theoretically dense formulations, and improving transitions between concepts. We also condensed several explanatory statements in the Discussion. These changes were intended solely to improve readability and narrative flow and do not affect the meaning, interpretation, or conclusions of the manuscript.We believe these revisions improve the clarity and accessibility of the manuscript while maintaining its conceptual depth.

The discussion of mentalizing has improved considerably following revision, particularly with the clarification that the RFQ_U primarily captures hypomentalizing rather than the full multidimensional construct of mentalizing. However, there remain a few instances where the manuscript refers broadly to “ineffective mentalizing” in ways that could be interpreted more expansively than the selected measure permits. A small amount of additional wording refinement may help ensure full alignment between theoretical claims and measurement specificity.

We particularly appreciate this comment, as conceptual clarity regarding mentalizing and its dimensions is a key focus of our broader research program. In response, we carefully reviewed the manuscript and replaced the term “ineffective mentalizing” with the more precise term “hypomentalizing” (or uncertainty about mental states, where appropriate) in sections directly referring to the present study, including the aims, results, and discussion. We retained broader terminology only in parts of the Introduction that discuss previous research in which the specific dimension of mentalizing under investigation was not clearly specified. In addition, we included a brief definition of hypomentalizing in the Introduction to clarify the specific aspect of mentalizing assessed in the present study.

Attachment Submitted filename: REV3_Response to Reviewers PLOS.pdf

# Editor report: Decision Letter 4

**DOI:** 10.1371/journal.pone.0353662.r009

**Authors:** Hong Wang Fung, Hong Wang Fung, Hong Wang Fung, Hong Wang Fung, Hong Wang Fung

28 Jun 2026

Betrayal Trauma and Adult Mental Health: The Role of Mentalizing and Dissociation

PONE-D-25-62663R4

Dear Dr. Jańczak,

We’re pleased to inform you that your manuscript has been judged scientifically suitable for publication and will be formally accepted for publication once it meets all outstanding technical requirements.

Within one week, you’ll receive an e-mail detailing the required amendments. When these have been addressed, you’ll receive a formal acceptance letter and your manuscript will be scheduled for publication.

An invoice will be generated when your article is formally accepted. Please note, if your institution has a publishing partnership with PLOS and your article meets the relevant criteria, all or part of your publication costs will be covered. Please make sure your user information is up-to-date by logging into Editorial Manager at Editorial Manager® and clicking the ‘Update My Information' link at the top of the page. For questions related to billing, please contact billing support.

If your institution or institutions have a press office, please notify them about your upcoming paper to help maximize its impact. If they’ll be preparing press materials, please inform our press team as soon as possible -- no later than 48 hours after receiving the formal acceptance. Your manuscript will remain under strict press embargo until 2 pm Eastern Time on the date of publication. For more information, please contact onepress@plos.org.

Kind regards,

Hong Wang Fung

Academic Editor

PLOS One

Additional Editor Comments (optional):

Thank you for addressing the comments from the two external reviewers.

Reviewers' comments:

# Editor report: Acceptance letter

**DOI:** 10.1371/journal.pone.0353662.r010

**Authors:** Hong Wang Fung, Hong Wang Fung, Hong Wang Fung, Hong Wang Fung, Hong Wang Fung

PONE-D-25-62663R4

PLOS One

Dear Dr. Jańczak,

I'm pleased to inform you that your manuscript has been deemed suitable for publication in PLOS One. Congratulations! Your manuscript is now being handed over to our production team.

At this stage, our production department will prepare your paper for publication. This includes ensuring the following:

* All references, tables, and figures are properly cited

* All relevant supporting information is included in the manuscript submission,

* There are no issues that prevent the paper from being properly typeset

You will receive further instructions from the production team, including instructions on how to review your proof when it is ready. Please keep in mind that we are working through a large volume of accepted articles, so please give us a few days to review your paper and let you know the next and final steps.

Lastly, if your institution or institutions have a press office, please let them know about your upcoming paper now to help maximize its impact. If they'll be preparing press materials, please inform our press team within the next 48 hours. Your manuscript will remain under strict press embargo until 2 pm Eastern Time on the date of publication. For more information, please contact onepress@plos.org.

You will receive an invoice from PLOS for your publication fee after your manuscript has reached the completed accept phase. If you receive an email requesting payment before acceptance or for any other service, this may be a phishing scheme. Learn how to identify phishing emails and protect your accounts at https://explore.plos.org/phishing.

If we can help with anything else, please email us at customercare@plos.org.

Thank you for submitting your work to PLOS ONE and supporting open access.

Kind regards,

PLOS ONE Editorial Office Staff

on behalf of

Dr. Hong Wang Fung

Academic Editor

PLOS One
